# Characterization of moose intestinal glycosphingolipids

**DOI:** 10.1007/s10719-015-9604-8

**Published:** 2015-06-24

**Authors:** Miralda Madar Johansson, Benjamin Dedic, Klara Lundholm, Filip Berner Branzell, Angela Barone, John Benktander, Susann Teneberg

**Affiliations:** Institute of Biomedicine, Department of Medical Biochemistry and Cell Biology, University of Gothenburg, P.O. Box 440, S-405 30 Gothenburg, Sweden

**Keywords:** Glycosphingolipid characterization, Mass spectrometry, Blood group antigens, SSEA-4, Sd^a^ determinant

## Abstract

As a part of a systematic investigation of the species-specific expression of glycosphingolipids, acid and non-acid glycosphingolipids were isolated from three small intestines and one large intestine of the moose (*Alces alces*). The glycosphingolipids were characterized by binding of monoclonal antibodies, lectins and bacteria in chromatogram binding assays, and by mass spectrometry. The non-acid fractions were complex mixtures, and all had glycosphingolipids belonging to the lacto- and neolactoseries (lactotriaosylceramide, lactotetraosylceramide, neolactotetraosylceramide, Galα3-Le^x^ hexaosylceramide, and lacto-neolactohexaosylceramide), globo-series (globotriaosylceramide and globotetraosylceramide), and isogloboseries (isoglobotriaosylceramide). Penta- and heptaglycosylceramides with terminal Galili determinants were also characterized. Furthermore, glycosphingolipids with terminal blood group O determinants (H triaosylceramide, H type 2 pentaosylceramide, H type 1 penta- and heptaosylceramide) were characterized in two of the moose small intestines, and in the one large intestine, while the third small intestine had glycosphingolipids with terminal blood group A determinants (A tetraosylceramide, A type 1 hexa- and octaosylceramide, A dodecaosylceramide). The acid glycosphingolipid fractions of moose small and large intestine contained sulfatide, and the gangliosides GM3, GD3, GD1a, GD1b, and also NeuGc and NeuAc variants of the Sd^a^ ganglioside and the sialyl-globopenta/SSEA-4 ganglioside. In humans, the NeuAc-globopenta/SSEA-4 ganglioside is a marker of embryonic and adult stem cells, and is also expressed in several human cancers. This is the first time sialyl-globopentaosylceramide/SSEA-4 has been characterized in a fully differentiated normal tissue, and also the first time NeuGc-globopentaosylceramide has been characterized.

**Electronic supplementary material:**

The online version of this article (doi:10.1007/s10719-015-9604-8) contains supplementary material, which is available to authorized users.

## Introduction

Glycosphingolipids are present in all vertebrate cell membranes and, due to their strategic position on the outer membrane, they can participate in a large number of recognition processes. The expression of glycosphingolipids differs between species, individuals, tissues and cells, and there are also developmental changes. This variable expression among species may be illustrated by the non-acid glycosphingolipids of the small intestine, where the mouse has mainly ganglio series compounds, the dog, cat, and rabbit mainly glycosphingolipids with neolacto/type 2 core, and the predominant complex glycosphingolipids of human, porcine and rat small intestine are based on lacto/type 1 core chains [1–3].

So far mainly the intestinal glycosphingolipids of domestic and laboratory animals have been characterized. In order to broaden the perspective, we have in this study isolated acid and non-acid glycosphingolipids from three small intestines and one large intestine of the moose (*Alces alces*). The glycosphingolipids were characterized by binding of monoclonal antibodies, lectins and bacteria in chromatogram binding assays, and by mass spectrometry. As in the human small intestine, the complex non-acid glycosphingolipids had mainly lacto/type 1 core chains. Glycosphingolipids with blood group A determinants were characterized in one of the small intestinal samples, while blood group H determinants were present in the non-acid glycosphingolipid fractions of the other two moose small intestines, and the large intestinal fraction. The acid fractions contained sulfatide and a complex mixture of NeuGc- and NeuAc-carrying gangliosides. Here, NeuGc and NeuAc variants of the sialyl-globopenta/SSEA-4 ganglioside and the Sd^a^ ganglioside were characterized, in addition to the more “ordinary” gangliosides as GM3, GD3, GD1a and GD1b.

## Materials and methods

### Glycosphingolipid preparations

The intestines from the three moose were gifts from the local hunters. After the animals were killed the intestines were taken out and washed carefully in a small river. The material was kept on ice until delivery at the laboratory, where it was frozen at −70 °C. The intestines were then lyophilized, and acid and non-acid glycosphingolipids were thereafter isolated as described [4]. Thus, the intestines were extracted in two steps in a Soxhlet apparatus with chloroform and methanol (2:1 and 1:9, by volume, respectively). The material thereby obtained was subjected to mild alkaline hydrolysis and dialysis, followed by separation on a silicic acid column. Acid and non-acid glycosphingolipid fractions were obtained by chromatography on a DEAE-cellulose column. In order to separate the non-acid glycosphingolipids from alkali-stable phospholipids, the non-acid fraction was acetylated and separated on a second silicic acid column, followed by deacetylation and dialysis. Final purifications were done by chromatographies on DEAE-cellulose and silicic acid columns.

Thereafter, 80 mg of the total non-acid glycosphingolipid fraction from moose I small intestine was separated on a silicic acid column eluted with increasing volumes of methanol in chloroform. Thereby one fraction containing monoglycosylceramides (denoted fraction N-I) and one fraction containing diglycosylceramides and more slow-migrating glycosphingolipids was obtained. The latter fraction (32.0 mg) was separated on an Iatrobeads (Iatrobeads 6RS-8060; Iatron Laboratories, Tokyo) column (10 g) eluted with increasing volumes of methanol in chloroform. This gave one mono- and diglycosylceramide fraction (7.4 mg; fraction N-II), and one fraction containing triglycosylceramides and more slow-migrating glycosphingolipids (19.0 mg). This fraction was separated on an Iatrobeads column (25 g) eluted with 60 × 1 ml chloroform/methanol/water 60:35:8 (by volume), followed by 10 ml chloroform/methanol/water 60:35:8, 20 ml chloroform/methanol/water 60:35:8 and finally 50 ml chloroform/methanol/water 60:35:8. Throughout the separation procedures aliquots of the fractions obtained were analyzed by thin-layer chromatography and anisaldehyde detection. The fractions were pooled according to their migration on thin-layer chromatograms giving five fractions denoted N-III (0.5 mg), N-IV (7.9 mg), N-V (3.6 mg), N-VI (3.0 mg), and N-VII (0.9 mg), respectively.

The non-acid glycosphingolipids of moose II small intestine were also separated by repeated chromatographies on Iatrobeads columns. Here we focused on isolation of compounds recognized by antibodies directed against the blood group A determinant. Thus, the fractions were pooled according to their migration on thin-layer chromatograms and their reactivity with the monoclonal anti-A antibodies. Finally, ten subfractions were collected (denoted fractions N2-I – N2-X). Fraction N2-IV had binding of anti-A antibodies in the tetraosylceramide region, fraction N2-VII had anti-A binding in the hexa- and octaosylceramide regions, while in fraction N2-IX the antibodies bound in octaosylceramide region and to a slow-migrating compound.

A similar strategy was used for separation of the total acid glycosphingolipid fractions from moose I small intestine. Here the acid glycosphingolipids (160 mg) were first separated on an Iatrobeads column (2 g) eluted with increasing volumes of methanol in chloroform. The glycosphingolipid fraction obtained (88.3 mg) was again separated on an Iatrobeads column (1 g) eluted with increasing volumes of methanol in chloroform. This gave one fraction (15.3 mg denoted fraction A-I) co-migrating with sulfatide on thin-layer chromatograms, one fraction (30.5 mg; fraction A-II) co-migrating with the GM3 ganglioside, and one fraction (20.6 mg) containing slow-migrating gangliosides. The latter fraction was separated on three subsequent Iatrobeads columns eluted with chloroform/methanol/water 60:35:8 (by volume). Aliquots of the fractions obtained were analyzed by thin-layer chromatography and detection with anisaldehyde and resorcinol. The fractions were pooled according to their migration on thin-layer chromatograms giving five fractions denoted A-III (0.5 mg), A-IV (1.1 mg), A-V (0.4 mg), A-VI (1.8 mg), and A-VII (0.5 mg), respectively.

Separation of the total acid and the total non-acid glycosphingolipid fractions from moose I large intestine was done in the same manner. Thus, the total acid or non-acid glycosphingolipid fractions were first separated on a silicic acid column eluted with increasing volumes of methanol in chloroform. This was followed by repeated chromatographies on Iatrobeads columns eluted with chloroform/methanol/water 60:35:8 (by volume).

### Reference glycosphingolipids

Total acid and non-acid glycosphingolipid fractions were isolated as described [4]. Individual glycosphingolipids were isolated by repeated chromatography on silicic acid columns and by HPLC, and identified by mass spectrometry ([5, 6] and ^1^H-NMR spectroscopy [7].

### Thin-layer chromatography

Thin-layer chromatography was done on aluminum- or glass-backed silica gel 60 high performance thin-layer chromatography plates (Merck, Darmstadt, Germany). Glycosphingolipid mixtures (20–40 μg) or pure glycosphingolipids (4 μg) were applied to the plates, and eluted with chloroform/methanol/water (60:35:8, by volume) as solvent system. Chemical detection was done with anisaldehyde [8], or the resorcinol reagent [9].

### Chromatogram binding assays

*Erythrina cristagalli* lectin was purchased from Vector Labs, and *Griffonia simplicifolia* IB4 lectin from Advanced Targeting Systems. Monoclonal anti-A (HE193), anti-B (HEB-29), and anti-H type 1 (17–206) antibodies were obtained from GeneTex/Abcam. Rabbit anti-mouse antibodies (Z0259) were from DakoCytomation Norden A/S, anti-globopenta/SSEA-3 (MC-631), and anti-sialyl-globopenta/SSEA-4 (MC-813-70) from eBioscience, anti-GD1a (GD1a-1) from Millipore, and anti-Lewis^x^ (P12) was from Calbiochem. Rabbit polyclonal anti-GM2 serum was purchased from Calbiochem, and goat anti-rabbit serum was from Aviva Systems Biology.

Binding of antibodies to glycosphingolipids on thin-layer chromatograms was done as described previously [10]. Dried chromatograms were dipped in diethylether/*n*-hexane (1:5 *v*/*v*) containing 0.5 % (*w*/*v*) polyisobutylmethacrylate for 1 min. To diminish background binding the chromatograms were blocked for 2 h at room temperature with phosphate-buffered saline (PBS, pH 7.3) containing 2 % (*w*/*v*) bovine serum albumin and 0.1 % (*w*/*v*) NaN_3_ (BSA/PBS). Suspensions of monoclonal antibodies diluted in BSA/PBS (the dilution used for each antibody is given in Table 1) were gently sprinkled over the chromatograms, followed by incubation for 2 h at room temperature. After washing with PBS followed a second 2 h incubation with ^125^I-labeled (labeled according to the IODO-GEN protocol of the manufacturer (Pierce, Rockford, IL)) rabbit anti-mouse antibodies, or goat anti-rabbit antibodies, diluted to 2 × 10^6^ cpm/ml in BSA/PBS. Finally, the plates were washed six times with PBS. Dried chromatograms were autoradiographed for 12–72 h using XAR-5 X-ray films (Eastman Kodak).

**Table 1:** Antibodies used in chromatogram binding assays

Antibodies	Clone	Manufacturer	Dilution	Isotype
Anti-blood group A	HE-193	GeneTex/Abcam	1:500	IgM
Anti-blood group B	HEB-29	GeneTex/Abcam	1:100	IgM
Anti-blood group H type 1	17-206	GeneTex/Abcam	1:100	IgG3
Anti-Globopenta/SSEA-3	MC-631	eBioscience	1:50	IgM
Anti-Lewis^x^/SSEA-1	P12	Calbiochem	1:200	IgM
Anti-Sialyl-globopenta/SSEA-4	MC-813-70	eBioscience	1:100	IgG3
Anti-GD1a	GD1a-1	Millipore	1:100	IgG1
Anti-GM2 (polyclonal)	–	Calbiochem	1:100	

Chromatogram binding assays with ^125^I-labeled *E. cristagalli* lectin, and *G. simplicifolia* IB4 lectin, were done as described [11]. Binding of ^35^S-labeled BabA expressing *H. pylori* strain J99 to glycosphingolipids on thin-layer chromatograms was done as described [12].

### Endoglycoceramidase digestion and LC-ESI/MS of oligosaccharides

Endoglycoceramidase II from *Rhodococcus* spp. (Takara Bio Europe S.A., Gennevilliers, France) was used for hydrolysis. Briefly, 50 μg of non-acid glycosphingolipids were resuspended in 100 μl 0.05 M sodium acetate buffer, pH 5.0, containing 120 μg sodium cholate, and sonicated briefly. Thereafter, 1 mU of endoglycoceramidase II was added and the mixture was incubated at 37 ° C for 48 h. The reaction was stopped by addition of chloroform/methanol/water to the final proportions 8:4:3 (by volume). The oligosaccharide-containing upper phase thus obtained was separated from detergent on a Sep-Pak QMA cartridge (Waters, Milford, MA). The eluant containing the oligosaccharides was dried under nitrogen and under vacuum.

The glycosphingolipid-derived oligosaccharides were resuspended in 50 μl of water and analyzed by LC-ESI/MS as described [6]. The oligosaccharides were separated on a column (200 × 0.180 mm) packed in-house with 5 μm porous graphite particles (Hypercarb, Thermo-Hypersil, Runcorn, UK). An autosampler, HTC-PAL (CTC Analytics AG, Zwingen, Switzerland) equipped with a cheminert valve (0.25 mm bore) and a 2 μl loop, was used for sample injection. An Agilent 1100 binary pump (Agilent technologies, Palo Alto, CA) delivered a flow of 250 μl/min, which was splitted down in an 1/16″ microvolume-T (0.15 mm bore) (Vici AG International, Schenkon, Switzerland) by a 50 cm × 50 μm i.d. fused silica capillary before the injector of the autosampler, allowing approximately 2–3 μl/min through the column. The oligosaccharides (3 μl) were injected on to the column and eluted with an acetonitrile gradient (A: 10 mM ammonium bicarbonate; B: 10 mM ammonium bicarbonate in 80 % acetonitrile). The gradient (0–45 % B) was eluted for 46 min, followed by a wash step with 100 % B, and equilibration of the column for 24 min. A 30 cm × 50 μm m i.d. fused silica capillary was used as transfer line to the ion source.

The saccharides were analyzed in negative ion mode on an LTQ linear quadrupole ion trap mass spectrometer (Thermo Electron, San José, CA). The IonMax standard ESI source on the LTQ mass spectrometer was equipped with a stainless steel needle kept at −3.5 kV. Compressed air was used as nebulizer gas. The heated capillary was kept at 270 °C, and the capillary voltage was −50 kV. Full-scan (*m/z* 380–2 000, 2 microscans, maximum 100 ms, target value of 30 000) was performed, followed by data dependent MS^2^ scans of the three most abundant ions in each scan (2 microscans, maximum 100 ms, target value of 10 000). The threshold for MS^2^ was set to 500 counts. Normalized collision energy was 35 %, and an isolation window of 3 u, an activation q = 0.25, and an activation time of 30 ms, was used. Data acquisition and processing were conducted with Xcalibur software (Version 2.0.7). Manual assignment of glycan sequences was done on the basis of knowledge of mammalian biosynthetic pathways, with the assistance of the Glycoworkbench tool (Version 2.1), and by comparison of retention times and MS^2^ spectra of oligosaccharides from reference glycosphingolipids [6].

### LC-ESI/MS of native glycosphingolipids

Glycosphingolipids were dissolved in methanol:acetonitrile in proportion 75:25 (by volume) and separated on a 200 × 0.150 mm column, packed in-house with 5 μM polyamine II particles (YMC Europe GmbH, Dinslaken, Germany). An autosampler, HTC-PAL equipped with a cheminert valve (0.25 mm bore) and a 2 μl loop, was used for sample injection. An Agilent 1100 binary pump delivered a flow of 250 μl/min, which was splitted down in an 1/16″ microvolume-T (0.15 mm bore) by a 50 cm × 50 μm i.d. fused silica capillary before the injector of the autosampler, allowing approximately 2–3 μl/min through the column. Samples were eluted with an aqueous gradient (A:100 % acetonitrile to B: 10 mM ammonium bicarbonate). The gradient (0–50 % B) was eluted for 40 min, followed by a wash step with 100 % B, and equilibration of the column for 20 min. The samples were analyzed in negative ion mode on a LTQ linear quadropole ion trap mass spectrometer, with an IonMax standard ESI source equipped with a stainless steel needle kept at −3.5 kV. Compressed air was used as nebulizer gas. The heated capillary was kept at 270 °C, and the capillary voltage was −50 kV. Full scan (*m/z* 500–1800, two microscan, maximum 100 ms, target value of 30,000) was performed, followed by data-dependent MS^2^ scans (two microscans, maximun 100 ms, target value of 10.000) with normalized collision energy of 35 %, isolation window of 2.5 units, activation q = 0.25 and activation time 30 ms). The threshold for MS^2^ was set to 500 counts. Data acquisition and processing were conducted with Xcalibur software (Version 2.0.7). Manual assignment of glycosphingolipid sequences was done with the assistance of the Glycoworkbench tool (Version 2.1), and by comparison of retention times and MS^2^ spectra of reference glycosphingolipids.

## Results

### Isolation of glycosphingolipids from moose intestines

**Fig. 1:**
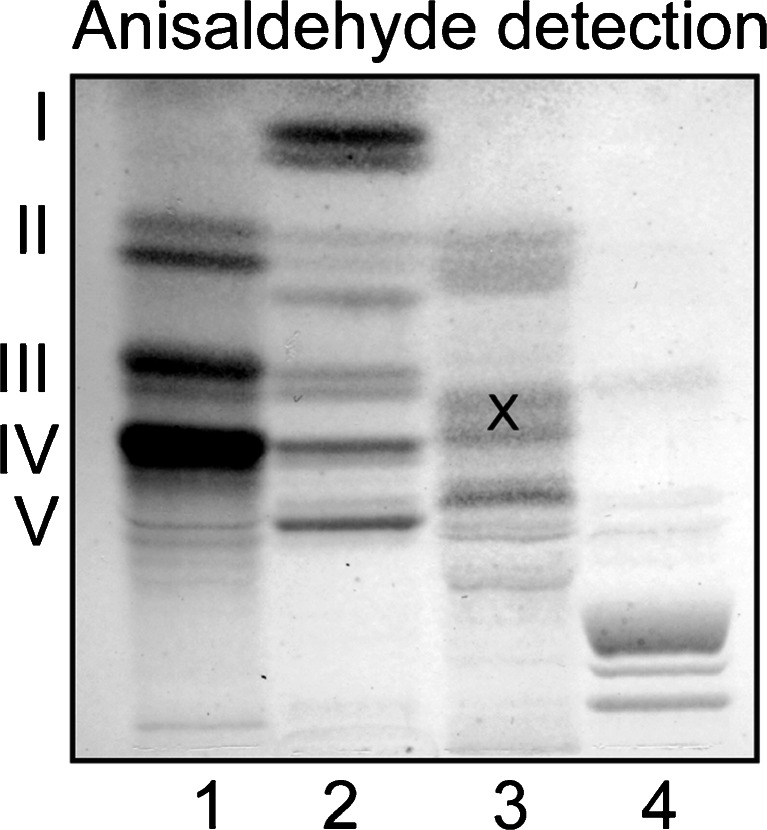
Thin-layer chromatogram of glycosphingolipids isolated from moose I small intestine. The chromatograms were eluted with chloroform/methanol/water 60:35:8 (by volume), and detection of glycosphingolipids was done with anisaldehyde. The lanes were: Lane 1, non-acid glycosphingolipids of human blood group AB erythrocytes, 40 μg; Lane 2, total non-acid glycosphingolipids of moose I small intestine, 40 μg; Lane 3, total acid glycosphingolipids of moose I small intestine, 40 μg; Lane 4, calf brain gangliosides, 40 μg. The Roman numbers to the left of panel A indicate the approximate number of carbohydrate units in the bands. The bands marked with an X in lane 3 are non-glycosphingolipid contaminants

**Fig. 2:**
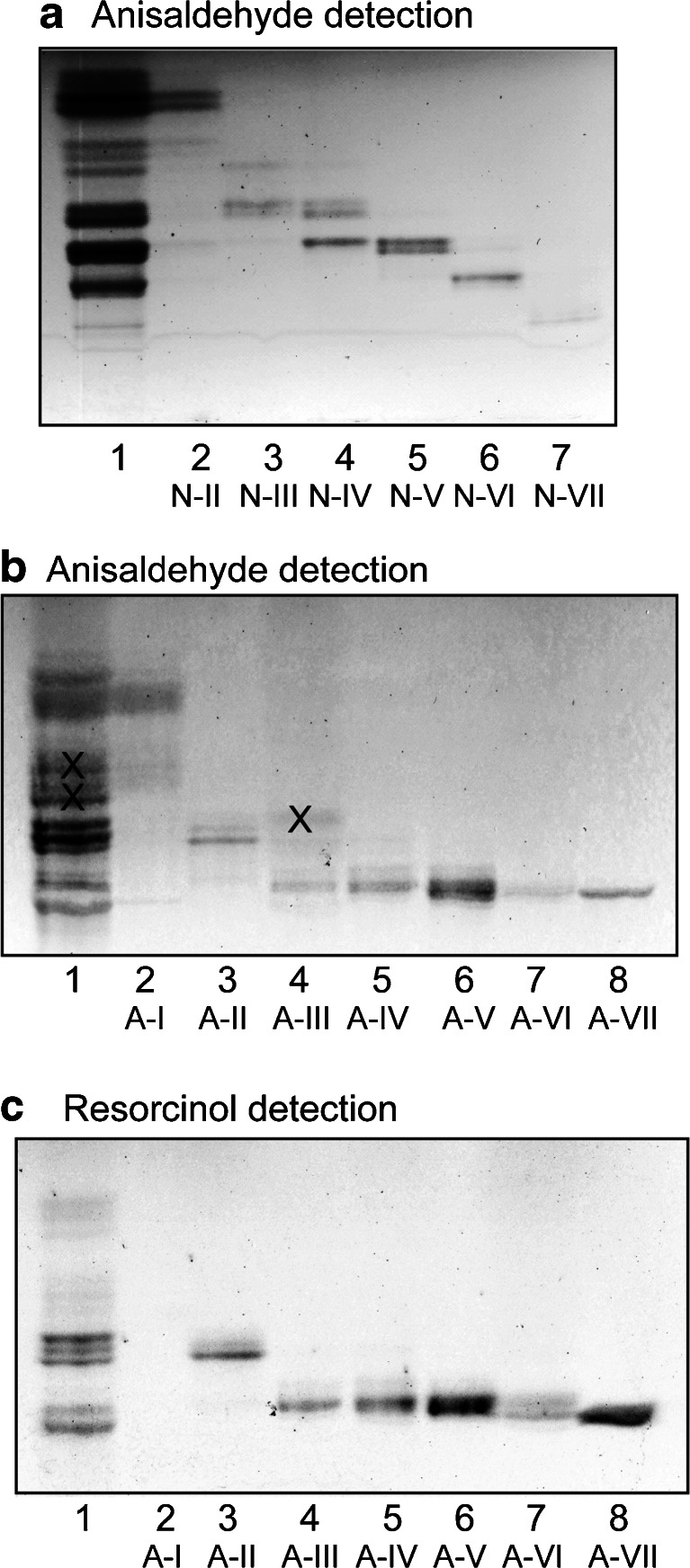
Separation of glycosphingolipids from moose I small intestine. Thin-layer chromatogram of non-acid (**a**) and acid (**b** and **c**) glycosphingolipid fractions isolated from moose I small intestine. The chromatograms were eluted with chloroform/methanol/water 60:35:8 (by volume), and detection of glycosphingolipids was done with anisaldehyde (**a** and **b**), and the resorcinol reagent (**c**). The lanes on (**a**) were: Lane 1, total non-acid glycosphingolipids of moose I small intestine, 40 μg; Lanes 2–7, fractions N-II – N-VII from moose I small intestine, 4 μg/lane. The lanes on (**b** and **c**) were: Lane 1, total acid glycosphingolipids of moose I small intestine, 40 μg; Lanes 2–8, fraction A-I – A-VII from moose I small intestine, 4 μg/lane. The bands marked with X on (**b**) are non-glycosphingolipid contaminants

Total acid and non-acid glycosphingolipids (exemplified in Fig. 1) were isolated from moose small and large intestines by standard procedures [4]. The acid and non-acid glycosphingolipid fractions from moose I and II small intestines, and moose I large intestine, were separated by chromatographies on silicic acid and Iatrobodies columns. In the case of moose I small intestine, this gave seven non-acid and seven acid subfractions. The non-acid subfractions contained compounds migrating as monoglycosylceramides (fraction N-I; not shown), mono- and diglycosylceramides (fraction N-II), di- and triglycosylceramides (fraction N-III), tri- and tetraglycosylceramides (fraction N-IV), tetraglycosylceramides (fraction N-V), pentaglycosylceramides (fraction N-VI), and heptaglycosylceramides (fraction N-VII) (Fig. 2a).

In the case of moose II small intestine ten subfractions were collected (denoted fractions N2-I–N2-X).

The seven acid subfractions from moose I small intestine are shown in Fig. 2b and c. The main compound of fraction A-I migrated relatively high on the thin-layer chromatogram, and was not stained by resorcinol. The other six fractions were resorcinol positive, indicating sialic acid-containing glycosphingolipids *i.e.,* gangliosides. Fraction A-II migrated at the level of the GM3 ganglioside, while the gangliosides of fraction A-III- A-VI were more slow-migrating.

#### Characterization of the non-acid glycosphingolipids of moose I small intestine

##### Chromatogram binding assays

**Fig. 3:**
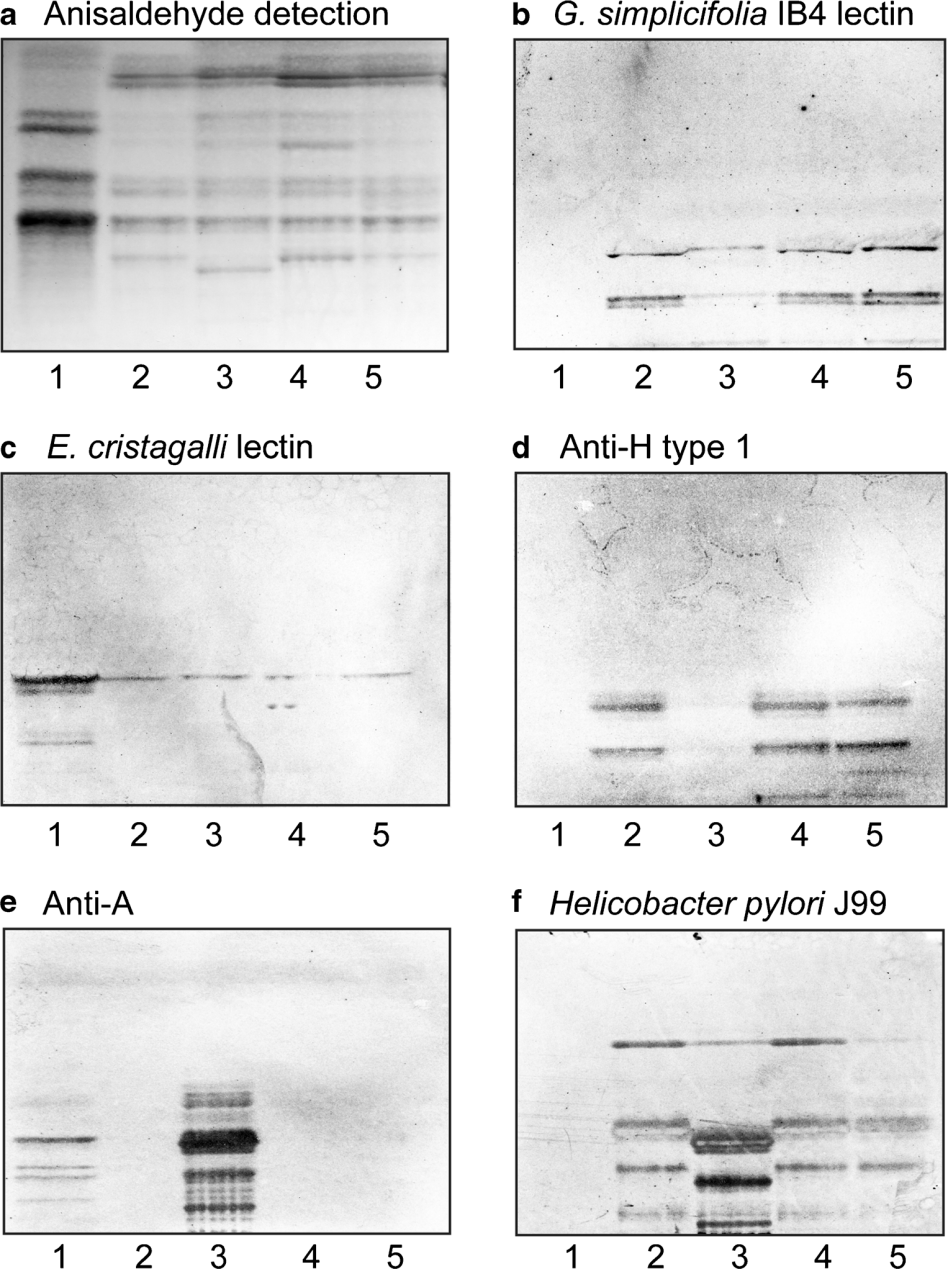
Comparison of the non-acid glycosphingolipid fractions from moose small and large intestine. Thin-layer chromatogram after detection with anisaldehyde (**a**), and autoradiograms obtained by binding of *G. simplicifolia* IB4 lectin (**b**), *E. cristagalli* lectin (**c**), the monoclonal anti-H type 1 antibody 17–206 (**d**), the monoclonal anti-A antibody HE193 (**e**), and BabA expressing *Helicobacter pylori* strain J99 (**f**). The glycosphingolipids were separated on aluminum-backed silica gel plates, using chloroform/methanol/water 60:35:8 (by volume) as solvent system, and the binding assays were performed as described under “Materials and methods”. Autoradiography was for 12 h. The lanes were: Lane 1, total non-acid glycosphingolipids of human blood group AB erythrocytes, 40 μg; Lane 2, total non-acid glycosphingolipids of moose I small intestine, 40 μg; Lane 3, total non-acid glycosphingolipids of moose II small intestine, 40 μg; Lane 4, total non-acid glycosphingolipids of moose III small intestine, 40 μg; Lane 5, total non-acid glycosphingolipids of moose I large intestine, 40 μg

At first, the binding of a number of carbohydrate binding ligands to the non-acid glycosphingolipids from moose intestines was tested. The Galα3 binding IB4 lectin from *G. simplicifolia* recognized compounds migrating in the penta- and heptaglycosylceramide regions in the non-acid fractions from the three moose small intestines and the one moose large intestine (Fig. 3b, lanes 2–5). The lectin from *E. cristagalli* binds to glycoconjugates with terminal Galβ4GlcNAc and Fucα2Galβ4GlcNAc sequences [11]. Binding of the *E. cristagalli* lectin in the tetraglycosylceramide region in the non-acid fractions from moose small and large intestines was obtained (Fig. 3c, lanes 2–5), indicating the presence of a Galβ4GlcNAc-terminated glycosphingolipid, *i.e.,* neolactotetraosylceramide (Galβ4GlcNAcβ3Galβ4Glcβ1Cer). The monoclonal antibodies directed against the blood group H type 1 epitope bound to compounds migrating in the penta- and heptaglycosylceramide regions in the non-acid fractions from the small intestine of moose I and III, and the large intestine of moose I (Fig. 3d, lanes 2, 4 and 5), indicating the presence of Fucα2Galβ3GlcNAc-terminated penta- and heptaglycosylceramides. However, no binding of the anti-H type 1 antibodies to the non-acid glycosphingolipid fraction of moose II small intestine was obtained (Fig. 3d, lane 3). Instead, in moose II small intestine fraction there was a distinct binding of monoclonal antibodies directed against blood group A determinant to compounds migrating as tetra- and hexaglycosylceramides, and also more slow-migrating glycosphingolipids (Fig. 3e, lane 3). The BabA adhesin of *Helicobacter pylori* strain J99 recognizes blood group A/B/H determinants on type 1 and type 4 core chains, in addition to binding to the Le^b^ determinant [12]. Upon binding of the J99 strain to the moose intestinal glycosphingolipids, the binding pattern obtained appeared as a merge of the binding obtained with the anti-H type 1 antibodies and the anti-A antibodies (Fig. 3f, lanes 2–5). In addition there was a binding in the diglycosylceramide region, most likely representing *H. pylori* lactosylceramide binding [13].

Binding of monoclonal antibodies directed against the blood group B and the globopenta/SSEA-3 epitopes to the non-acid glycosphingolipids from moose intestines was also tested. However, no binding was observed (data not shown).

**Fig. 4:**
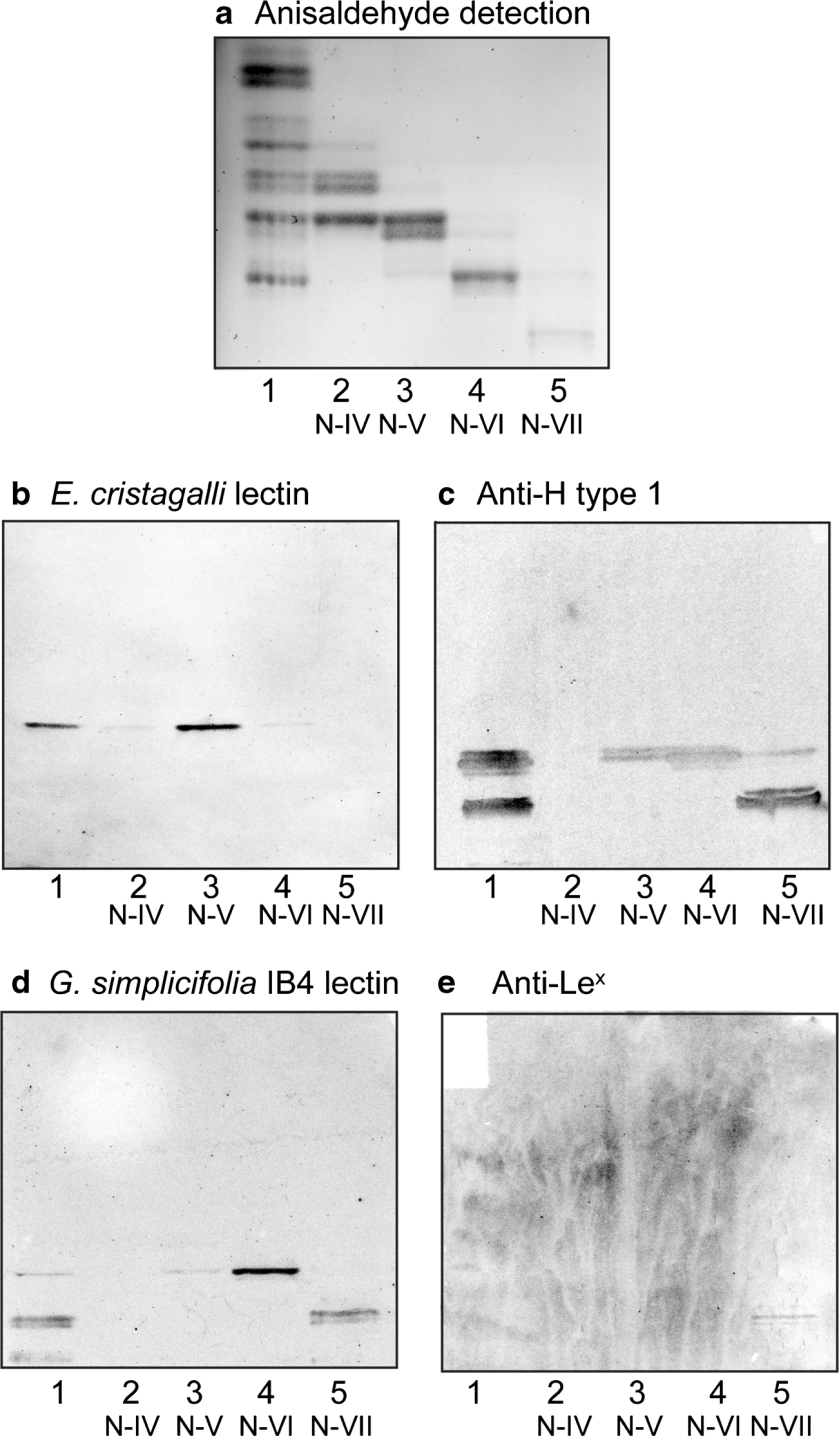
Binding of lectins and monoclonal antibodies to the non-acid glycosphingolipid subfractions isolated from moose I small intestine. Thin-layer chromatogram after detection with anisaldehyde (**a**), and autoradiograms obtained by binding of *E. cristagalli* lectin (**b**), the monoclonal anti-H type 1 antibody 17–206 (**c**), *G. simplicifolia* IB4 lectin (**d**), and the monoclonal anti-Lewis^x^ antibody P12 (**e**). The glycosphingolipids were separated on aluminum-backed silica gel plates, using chloroform/methanol/water 60:35:8 (by volume) as solvent system, and the binding assays were performed as described under “ Materials and methods”. Autoradiography was for 12 h. The lanes were: Lane 1, total non-acid glycosphingolipids of moose I small intestine, 40 μg; Lanes 2–5, fraction N-IV – N-VII from moose I small intestine, 4 μg/lane

Next, the binding of lectins and monoclonal antibodies to the non-acid subfractions isolated from the small intestine of moose I was evaluated. Here the *E. cristagalli* lectin bound to fraction N-V (Fig. 4b), while the anti H type 1 binding activity was found in fractions N-V and N-VI (pentaglycosylceramide region), and fraction N-VII (heptaglycosylceramide region) (Fig. 4c). Binding to fraction N-VI (pentaglycosylceramide region), and fraction N-VII (heptaglycosylceramide region) was also obtained with the *G. simplicifolia* IB4 lectin (Fig. 4d). A weak but distinct binding of the anti Lewis^x^ antibodies to fraction N-VII (heptaglycosylceramide region) was also observed (Fig. 4e).

#### Mass spectrometry

##### Fractions N-I and N-II

The base chromatogram from ESI/MS of fraction N-I had a number of molecular ions corresponding to monoglycosylceramides, while the molecular ions in the base chromatogram of fraction N-II indicated mono- and diglycosylceramides (data not shown). The major monoglycosylceramide ions were found at *m/z* 816 and *m/z* 844, corresponding to the species with t18:0-h22:0 and t18:0-h24:0, respectively. The diglycosylceramide ions were found at *m/z* 860, *m/z* 876, *m/z* 894, *m/z* 978, and *m/z* 1006, corresponding to the species with d18:1–16:0, d18:1-h16:0, t18:0-h16:0, t18:0-h22:0 and t18:0-h24:0, respectively.

##### Fractions N-III to N-VII

Fractions N-III to N-VII were hydrolyzed with *Rhodococcus* endoglycoceramidase II, and the oligosaccharides thereby obtained were analyzed by LC-ESI/MS using graphitized carbon columns. This gives a resolution of isomeric oligosaccharides, and the carbohydrate sequence may be deduced from series of C-type fragment ions obtained by MS^2^ [6]. MS^2^ spectra of oligosaccharides with a Hex or HexNAc substituted at C-4 have diagnostic cross-ring ^0,2^A-type fragment ions, which allow differentiation of linkage positions.

**Fig. 5:**
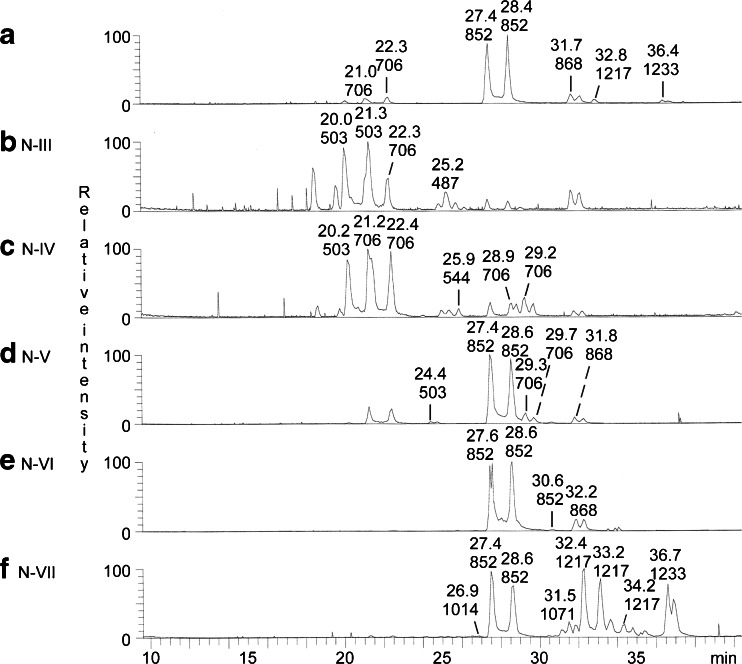
LC-ESI/MS of the oligosaccharides obtained by digestion of non-acid glycosphingolipids from moose I small intestine with *Rhodococcus* endoglycoceramidase II. **a** Base peak chromatogram from LC-ESI/MS of the oligosaccharides obtained by digestion of the total non-acid glycosphingolipid fraction from moose I small intestine. **b** Base peak chromatogram from LC-ESI/MS of the oligosaccharides derived from fraction N-III. **c** Base peak chromatogram from LC-ESI/MS of the oligosaccharides derived from fraction N-IV. **d** Base peak chromatogram from LC-ESI/MS of the oligosaccharides derived from fraction N-V. **e** Base peak chromatogram from LC-ESI/MS of the oligosaccharides derived from fraction N-VI. **f** Base peak chromatogram from LC-ESI/MS of the oligosaccharides derived from fraction N-VII

The base peak chromatogram from LC-ESI/MS of the oligosaccharides obtained from the total non-acid glycosphingolipid fraction from moose I small intestine is shown in Fig. 5a. Molecular ions corresponding to oligosaccharides ranging from trisaccharides (detected as [M-H^+^]^−^ ions at *m/z* 503) to heptasaccharides (detected as [M-H^+^]^−^ ions at *m/z* 1217 and 1233) were found. In addition, a number of minor oligosaccharides were detected in the mass chromatograms from LC-ESI/MS of fractions N-III to N-VII (Fig. 5b–f).

##### Fractions N-III, N-IV and N-V

MS^2^ of the molecular ions in the base peak chromatograms of fractions N-III and N-IV (Fig. 5b and c) allowed a tentative identification of a number of glycosphingolipid derived oligosaccharides. The MS^2^ data are summarized in Table 2. Thus, a globo trisaccharide (Galα4Galβ4Glc), a globo tetrasaccharide (GalNAcβ3Galα4Galβ4Glc), and an H trisaccharide (Fucα2Galβ4Glc) were present in fraction N-III, while fraction N-IV had a globo trisaccharide, a globo tetrasaccharide, a lacto trisaccharide (GlcNAcβ3Galβ4Glc), a lacto tetrasaccharide (Galβ3GlcNAcβ3Galβ4Glc), and a neolacto tetrasaccharide (Galβ4GlcNAcβ3Galβ4Glc).

**Table 2:** LC-ESI/MS of the oligosaccharides obtained by digestion of the non-acid fractions N-III and N-IV from moose I small intestine with *Rhodococcus* endoglycoceramidase II. Summary of MS^2^ data

Fraction	[M-H^+^]^−^ ion	RT (min)	C ions (*m/z*)	^0,2^A ions (*m/z*)^a^	Carbohydrate sequence	Tentative oligosaccharide
N-III	*m/z* 503	20.0/21.3	C_1_ 179; C_2_ 341	^0,2^A_2_ 281	Hex-4Hex-4Hex	Galα4Galβ4Glc
	*m/z* 706	22.3	C_1_ 220; C_2_ 382; C_3_ 544	^0,2^A_3_ 484	HexNAc-Hex-4Hex-4Hex	GalNAcβ3Galα4Galβ4Glc
	*m/z* 487	25.2	C_1_ 163; C_2_ 325	–	Fuc-Hex-4Hex	Fucα2Galβ4Glc
N-IV	*m/z* 503	20.2	C_1_ 179; C_2_ 341	^0,2^A_2_ 281	Hex-4Hex-4Hex	Galα4Galβ4Glc
	*m/z* 706	21.2/22.4	C_1_ 220; C_2_ 382; C_3_ 544	^0,2^A_3_ 484	HexNAc-Hex-4Hex-4Hex	GalNAcβ3Galα4Galβ4Glc
	*m/z* 544	25.9	C_1_ at *m/z* 220, C_2_ at *m/z* 382	–	HexNAc-Hex-4Hex	GlcNAcβ3Galβ4Glc
	*m/z* 706	28.9	C_2_ 382; C_3_ at *m/z* 544	–	Hex-HexNAc-Hex-4Hex	Galβ3GlcNAcβ3Galβ4Glc
	*m/z* 706	29.2	C_2_ 382; C_3_ at *m/z* 544	^0,2^A_2_ 281	Hex-4HexNAc-Hex-4Hex	Galβ4GlcNAcβ3Galβ4Glc

^a^The ^0,2^A ions derived from the lactose unit at the reducing end are not given

The base peak chromatogram of the oligosaccharides derived from fraction N-V (Fig. 5d) showed that this fraction was a mixture of compounds found in fraction N-IV and fraction N-VI. The only unique feature was the ion at *m/z* 503 eluting at 24.4 min. Here MS^2^ gave a B_1_ ion at *m/z* 161, and a C-type ion series (C_1_ at *m/z* 179 and C_2_ at *m/z* 341), demonstrating a Hex-Hex-Hex sequence. There was no ^0,2^A_2_ fragment ion at *m/z* 281, suggesting that the internal Hex was substituted at C-3. Taken together, these spectral features indicated the presence of a isoglobotrisaccharide (Galα3Galβ4Glc).

##### Fraction N-VI

**Fig. 6:**
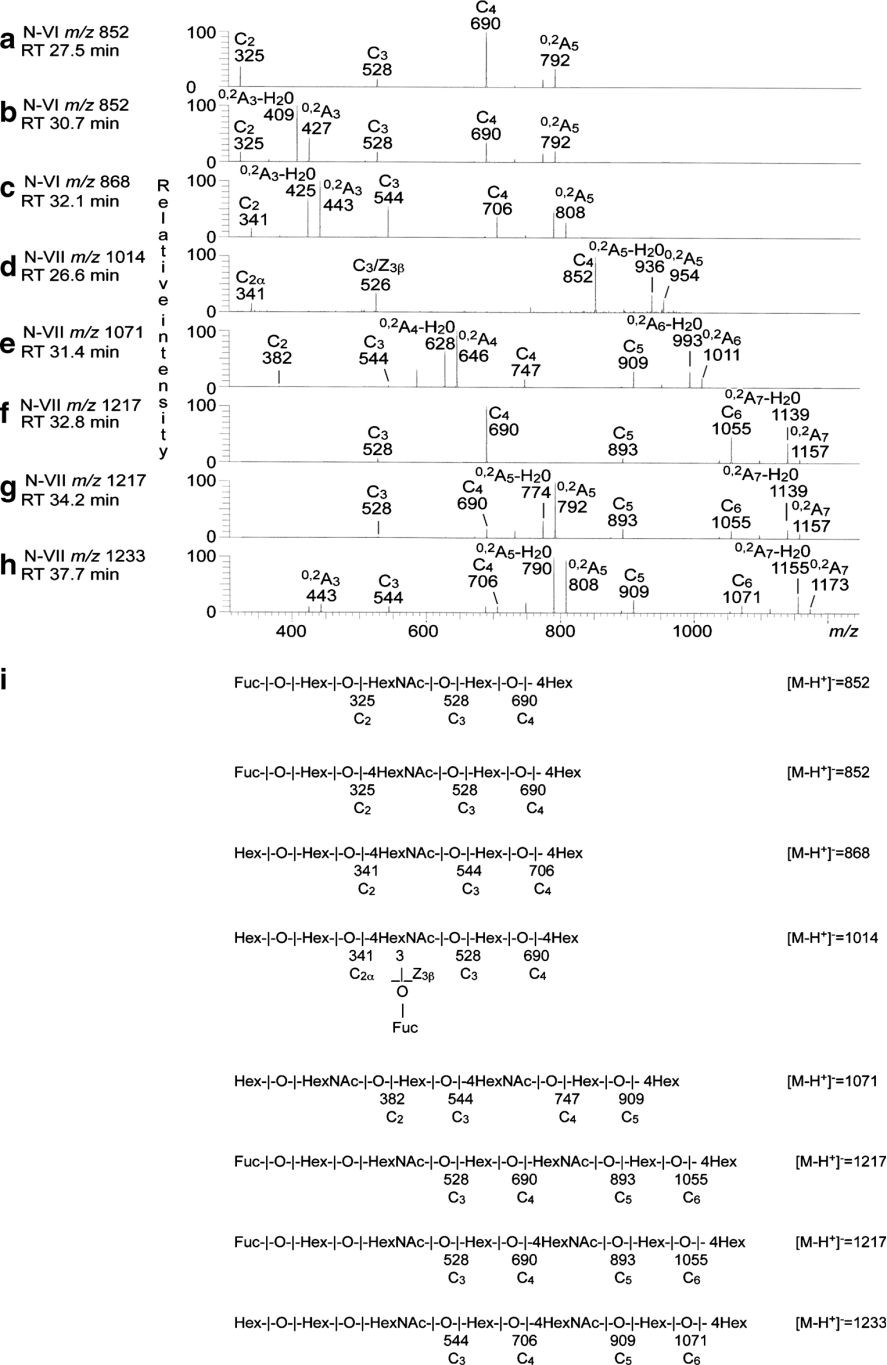
LC-ESI/MS of the oligosaccharides obtained by digestion of non-acid glycosphingolipids (fraction N-VI and N-VII) from moose I small intestine with *Rhodococcus* endoglycoceramidase II. **a** MS^2^ of the ion at *m/z* 852 (retention time 27.5 min) from LC-ESI/MS of fraction N-VI. **b** MS^2^ of the ion at *m/z* 852 (retention time 30.7 min) from LC-ESI/MS of fraction N-VI. **c** MS^2^ of the ion at *m/z* 868 (retention time 32.1 min) from LC-ESI/MS of fraction N-VI. **d** MS^2^ of the ion at *m/z* 1014 (retention time 26.6 min) from LC-ESI/MS of fraction N-VII. **e** MS^2^ of the ion at *m/z* 1071 (retention time 31.4 min) from LC-ESI/MS of fraction N-VII. **f** MS^2^ of the ion at *m/z* 1217 (retention time 32.8 min) from LC-ESI/MS of fraction N-VII. **g** MS^2^ of the ion at *m/z* 1217 (retention time 34.2 min) from LC-ESI/MS of fraction N-VII. **h** MS^2^ of the ion at *m/z* 1233 (retention time 37.7 min) from LC-ESI/MS of fraction N-VII. **i** Interpretation formulas showing the deduced carbohydrate sequences

The base peak chromatogram of fraction N-VI (Fig. 5e) had two molecular ions at *m/z* 852, one major eluting at 27.6–28.6 min and one minor eluting at 30.6 min. MS^2^ gave in both cases a series of C type fragment ions (C_2_ at *m/z* 325, C_3_ at *m/z* 528 and C_4_ at *m/z* 690) identifying a Fuc-Hex-HexNAc-Hex-Hex oligosaccharide (Fig. 6a and b). The MS^2^ spectrum of the minor ion eluting at 30.6 min (Fig. 6b) also had a prominent ^0,2^A_3_-H_2_O fragment ion at *m/z* 409 and a ^0,2^A_3_ fragment ion at *m/z* 427, characteristic for 4-substituted HexNAc, *i.e.,* type 2 carbohydrate chains. The features of the two spectra were very similar to those of reference H type 1 and H type 2 pentasaccharides, respectively [6], which allowed identification of one Fucα2Galβ3GlcNAcβ3Galβ4Glc saccharide (major ion) and one Fucα2Galβ4GlcNAcβ3Galβ4Glc saccharide (minor ion).

A molecular ion at *m/z* 868 was also present in the base peak chromatogram of fraction N-VI (Fig. 5e). Here, MS^2^ (Fig. 6c) gave a tentative identification of a Galα3Galβ4GlcNAc-terminated pentasaccharide (Galα3Galβ4GlcNAcβ3Galβ4Glc). This was concluded from the C-type fragment ion series (C_2_ at *m/z* 341, C_3_ at *m/z* 544, and C_4_ at *m/z* 706) identifying a Hex-Hex-HexNAc-Hex-Hex sequence, and the ^0,2^A_2_-H_2_O fragment ion at *m/z* 425, and the ^0,2^A_3_ fragment ion at *m/z* 443, demonstrating a substitution of the HexNAc at C-4, *i.e.,* a type 2 chain.

##### Fraction N-VII

The base peak chromatogram of the oligosaccharides obtained from fraction N-VII showed that this fraction was a complex mixture (Fig. 5f). Also here an H type 1 pentasaccharide was characterized by MS^2^ of the ion at *m/z* 852.

MS^2^ of oligosaccharides with terminal Le^x^ (Galβ4(Fucα3)GlcNAc-) sequence gives a fragment ion at *m/z* 364. This fragment ion is obtained by double glycosidic cleavage of the 3-linked branch at C_3_ and Z_3β_, and is characteristic for an internal 4-linked GlcNAc substituted with a Fuc at 3-position [14]. The base peak chromatogram of fraction N-VII (Fig. 5f) had a minor ion at *m/z* 1014, corresponding to an oligosaccharide with one Fuc, one HexNAc and four Hex. MS^2^ (Fig. 6d) gave an ion at *m/z* 526, which along with the C_2_ ion at *m/z* 341 denoting a terminal Hex-Hex sequence, indicated a Hex-Galβ4(Fucα3)GlcNAc terminal. Furthermore, there was a C_4_ ion at m/z 852. Taken together, these spectral features indicated the presence of a Galα3-Le^x^ hexasaccharide (Galα3Galβ4(Fucα3)GlcNAcβ3Galβ4Glc), previously characterized among porcine kidney glycosphingolipids [15].

The base chromatogram of fraction N-VII (Fig. 5f) also had a molecular ion at *m/z* 1071, and MS^2^ of this ion (Fig. 6e) gave a preliminary identification of a lacto-neolacto hexasaccharide (Galβ3GlcNAcβ3Galβ4GlcNAcβ3Galβ4Glc). This was concluded from the C-type fragment ions (C_2_ at *m/z* 382, C_3_ at *m/z* 544, C_4_ at *m/z* 747, and C_5_ at *m/z* 909) identifying a Hex-HexNAc-Hex-HexNAc-Hex-Hex sequence, along with the prominent ^0,2^A_4_-H_2_O fragment ion at *m/z* 628, and the ^0,2^A_4_ fragment ion at *m/z* 646, demonstrating 4-substitution of the HexNAc at the reducing end. MS^3^ of *m/z* 646 gave no ^0,2^A_2_ fragment ion at *m/z* 281, indicating that the penultimate HexNAc was substituted at C-3 (data not shown). The absence of binding of the Galβ4GlcNAc-recognizing lectin from *E. cristagalli* to fraction N-VII (Fig. 4b, lane 5) is consistent with this suggestion.

The two molecular ions at *m/z* 1217 in the base peak chromatogram of fraction N-VII (Fig. 5f) eluted at 32.4–33.2 min and 34.2 min, respectively. Here the MS^2^ spectra obtained were very similar (Fig. 6f and g), both having C-type ion series (C_3_ at *m/z* 528, C_4_ at *m/z* 690, C_5_ at *m/z* 893, and C_6_ at *m/z* 1055). MS^3^ of *m/z* 690 gave in both cases a prominent C_2_ ion at *m/z* 325, demonstrating a terminal Fuc-Hex sequence (data not shown), and taken together this demonstrated a heptasaccharide with Fuc-Hex-HexNAc-Hex-HexNAc-Hex-Hex sequence. In the MS^2^ spectrum of the ion eluting at 34.2 min there was also a ^0,2^A_5_-H_2_O ion at *m/z* 774, and a ^0,2^A_5_ ion at *m/z* 792, demonstrating that the reducing end HexNAc was substituted at C-4 (Fig. 6g). Furthermore, upon closer inspection of the low molecular range a ^0,2^A_3_-H_2_O ion at *m/z* 409, and a ^0,2^A_5_ ion at *m/z* 427, were found, demonstrating 4-substitution also of the first HexNAc (data not shown). Taken together these spectral features allowed tentative identification of one H type 1 heptasaccharide (Fucα2Galβ3GlcNAcβ3Galβ3GlcNAcβ3Galβ4Glc) and one H type 2 heptasaccharide (Fucα2Galβ4GlcNAcβ3Galβ4GlcNAcβ3Galβ4Glc).

Finally, a heptasaccharide with type 2 core chain and Hex-Hex-HexNAc-Hex-HexNAc-Hex-Hex sequence was suggested by MS^2^ of the ion at *m/z* 1233 of fraction N-VII (Fig. 6h). This was concluded from the C-type ion series (C_3_ at *m/z* 544, C_4_ at *m/z* 706, C_5_ at *m/z* 909, and C_6_ at *m/z* 1071), along with the ^0,2^A_3_ fragment ion at *m/z* 443, and the ^0,2^A_5_-H_2_O fragment ion at *m/z* 790, and the ^0,2^A_5_ fragment ion at *m/z* 808, demonstrating that the two HexNAcs were substituted at C-4. Upon MS^3^ of *m/z* 706 a prominent C_2_ ion at *m/z* 341 was observed, demonstrating a terminal Hex-Hex sequence (data not shown). Thus, these spectral features suggested a Galα3Galβ4GlcNAc-terminated heptasaccharide (Galα3Galβ4GlcNAcβ3Galβ4GlcNAcβ3Galβ4Glc).

In summary, chromatogram binding assays and mass spectrometry allowed identification of a number of non-acid glycosphingolipids of moose I small intestine, *i.e.,* mono- and dihexosylceramides, globotriaosylceramide, lactotriaosylceramide, isoglobotriaosylceramide, blood group H triaosylceramide, globotetraosylceramide, lactotetraosylceramide, neolactotetraosylceramide, blood group H type 1 pentaosylceramide, blood group H type 2 pentaosylceramide, Galili pentaosylceramide, Galα3-Le^x^ hexaosylceramide, lacto-neolactohexaosylceramide, H type 1 heptaosylceramide, H type 2 heptaosylceramide and Galili heptaosylceramide, were identified.

#### Characterization of the non-acid glycosphingolipids of moose II small intestine

**Fig. 7:**
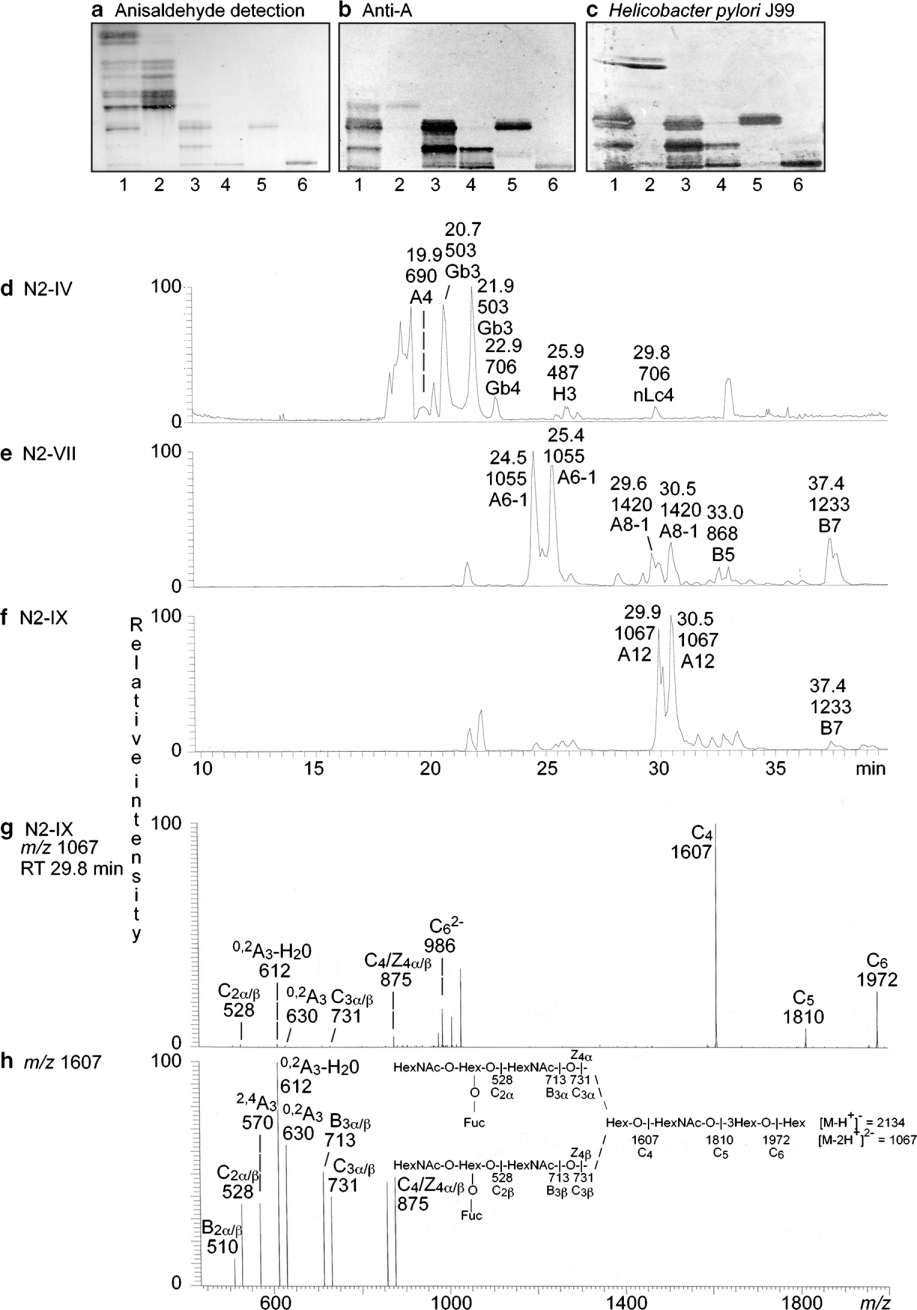
Characterization of the non-acid glycosphingolipids from moose II small intestine. Thin-layer chromatogram after detection with anisaldehyde (**a**), and autoradiograms obtained by the monoclonal anti-A antibody HE193 (**b**), and BabA expressing *Helicobacter pylori* strain J99 (**c**). The glycosphingolipids were separated on aluminum-backed silica gel plates, using chloroform/methanol/water 60:35:8 (by volume) as solvent system, and the binding assays were performed as described under “ Materials and methods”. Autoradiography was for 12 h. The lanes were: Lane 1, total non-acid glycosphingolipids of moose II small intestine, 40 μg; Lane 2, fraction N2-IV from moose II small intestine, 4 μg; Lane 3, fraction N2-VII from moose II small intestine, 4 μg; Lane 4, fraction N2-IX from moose II small intestine, 4 μg; Lane 5, reference A type 1 hexaosylceramide (GalNAcα3(Fucα2)Galβ3GlcNAcβ3Galβ4Glcβ1Cer), 4 μg; Lane 6, reference A type 1 dodecaosylceramide (GalNAcα3(Fucα2)Galβ3GlcNAcβ6(GalNAcα3(Fucα2)Galβ3GlcNAcβ3)Galβ3GlcNAcβ3Galβ4Glcβ1Cer), 4 μg. **d** Base peak chromatogram from LC-ESI/MS of the oligosaccharides obtained by digestion of fraction N2-IV from moose II small intestine with *Rhodococcus* endoglycoceramidase II. **e** Base peak chromatogram from LC-ESI/MS of the oligosaccharides obtained by digestion of fraction N2-VII from moose II small intestine with *Rhodococcus* endoglycoceramidase II. **f** Base peak chromatogram from LC-ESI/MS of the oligosaccharides obtained by digestion of fraction N2-IX from moose II small intestine with *Rhodococcus* endoglycoceramidase II. The identification of individual glycosphingolipid-derived oligosaccharides given in D-F was based on their determined molecular masses and subsequent MS^2^ sequencing. A4, GalNAcα3(Fucα2)Galβ4Glc; Gb3, Galα4Galβ4Glc; Gb4, GalNAcβ3Galα4Galβ4Glc; H3, Fucα2Galβ4Glc; nLc4, Galβ4GlcNAcβ3Galβ4Glc; A6-1, GalNAcα3(Fucα2)Galβ3GlcNAcβ3Galβ4Glc; A8-1, GalNAcα3(Fucα2)Galβ3GlcNAcβ3Galβ3GlcNAcβ3Galβ4Glc; B5, Galα3Galβ4GlcNAcβ3Galβ4Glc; B7, Galα3Galβ4GlcNAcβ3Galβ4GlcNAcβ3Galβ4Glc; A12, GalNAcα3(Fucα2)Galβ3GlcNAcβ6(GalNAcα3(Fucα2)Galβ3GlcNAcβ3)Galβ3GlcNAcβ3Galβ4Glc. **g** MS^2^ of the ion at *m/z* 1067 (retention time 29.8 min) from LC-ESI/MS of fraction N2-IX. **h** MS^3^ spectrum of the ion at *m/z* 1607. The interpretation formula shows the deduced carbohydrate sequence

In the case of moose II small intestine we focused on characterization of the major compounds recognized by antibodies directed against the blood group A determinant. To this end the total non-acid fraction was separated into ten fractions, and by binding of anti-A antibodies, and BabA expressing *Helicobacter pylori* strain J99 (Fig. 7b and c, lanes 2–4), three fractions were selected for structural characterization. In the first fraction (denoted fraction N2-IV) the anti-A antibodies bound in the tetraosylceramide region (Fig. 7b). The second fraction (denoted fraction N2-VII) had anti-A binding in the hexa- and octaosylceramide regions, while in the third fraction (denoted fraction N2-IX) the antibodies bound in octaosylceramide region and to a slow-migrating compound. The compounds migrating in the hexa- and octaosylceramide regions, and the slow-migrating compound, were also recognized by the *H. pylori* strain J99 with generalist BabA [16] (Fig. 7c).

##### Fraction N2-IV

The base peak chromatogram from LC-ESI/MS of the oligosaccharides of fraction N2-IV (Fig. 7d; Supplementary Figure 1A) had a number of molecular ions at *m/z* 503, *m/z* 706 and *m/z* 487. By MS^2^ of these ions allowed identification of globo trisaccharide, globo tetrasaccharide, an H trisaccharide, and a neolacto tetrasaccharide, as in the small intestine of moose I above.

In addition there was a minor molecular ion at *m/z* 690, and the MS^2^ spectrum obtained of this ion (Supplementary Figure 1C) demonstrated a blood group A tetrasaccharide (GalNAcα3(Fucα2)Galβ4Glc). This was concluded from the C-type fragment ions (C_1α_ at *m/z* 220 and C_2_ at *m/z* 528) identifying a HexNAc-(Fuc-)Hex-Hex sequence, along with the binding of anti-A antibodies in the tetraglycosylceramide region of this fraction (Fig. 7b, lane 2). The ^0,2^A_3_ fragment ion at *m/z* 630, and the ^0,2^A_2_-H_2_O fragment ion at *m/z* 612, were derived from cross-ring cleavage of the 4-substituted Glc of the lactose unit at the reducing end.

##### Fraction N2-VII

Molecular ions at *m/z* 1055, *m/z* 1420, *m/z* 868 and *m/z* 1233 were present in the base peak chromatogram of fraction N2-VII (Fig. 7e; Supplementary Figure 1B). As above, a Galili penta- and heptasaccharide were identified by MS^2^ of the ions at *m/z* 868 and *m/z* 1233, respectively.

MS^2^ of the ion at *m/z* 1055 (Supplementary Figure 1D) gave a series of C-type fragment ions (C_2_ at *m/z* 528, C_3_ at *m/z* 731, and C_4_ at *m/z* 893) indicating a HexNAc-(Fuc)Hex-HexNAc-Hex-Hex sequence. There was no ^0,2^A_3_ fragment ion at *m/z* 630, suggesting that the internal HexNAc was substituted at C3, *i.e.,* a type 1 core chain. This MS^2^ spectrum was very similar to the MS^2^ spectrum of reference blood group A type 1 hexasaccharide [6]. Thus, a hexasaccharide with HexNAc-(Fuc)Hex-HexNAc-Hex-Hex sequence and an internal HexNAc substituted at C-3 was indicated, most likely a blood group A type 1 hexasaccharide (GalNAcα3(Fucα2)Galβ3GlcNAcβ3Galβ4Glc).

The same series of C-type fragment ions (C_2_ at *m/z* 528, C_3_ at *m/z* 731, and C_4_ at *m/z* 893) was obtained by MS^2^ of the ion at *m/z* 1420 (Supplementary Figure 1E). In addition, there was a C_5_ ion at *m/z* 1096 and a C_6_ ion at *m/z* 1258. Taken together this demonstrated a HexNAc-(Fuc)Hex-HexNAc-Hex-HexNAc-Hex-Hex sequence. Type 1 core chain was indicated by the absence of ^0,2^A_3_ and ^0,2^A_5_ fragment ions. Thus, a blood group A type 1 octasaccharide (GalNAcα3(Fucα2)Galβ3GlcNAcβ3Galβ3GlcNAcβ3Galβ4Glc) was tentatively identified.

##### Fraction N2-IX

LC-ESI/MS of the oligosaccharides obtained by hydrolysis of fraction N2-IX (Fig. 7f) gave a major [M-2H^+^]^2−^ ion at *m/z* 1067, corresponding to a [M-H^+^]^−^ ion at *m/z* 2134, demonstrating a dodecasaccharide with two Fuc, five HexNAc and five Hex. MS^2^ of *m/z* 1067 gave a weak lower mass region (Fig. 7g). However, there was a C_2_ ion at *m/z* 528 and a C_3_ ion at *m/z* 731, indicating a terminal HexNAc-(Fuc-)Hex-HexNAc sequence. In addition, there were intense C type ions at *m/z* 1607, *m/z* 1810, and *m/z* 1972. The MS^3^ spectrum of the ion at *m/z* 1607 also had a C_2_ ion at *m/z* 528 and a C_3_ ion at *m/z* 731 (Fig. 7h). This spectrum was dominated by a ^0,2^A_3_ ion at *m/z* 630, and a ^0,2^A_3_-H_2_O ion at *m/z* 612, which together with the C_2_ ion at *m/z* 528 and a C_3_ ion at *m/z* 731, identified indicating a terminal HexNAc-(Fuc-)Hex-HexNAc sequence sequence with 4-substitution of the internal HexNAc, *i.e.,* a type 2 core chain. Finally, a C_1_ ion at *m/z* 220, confirming a terminal HexNAc, was obtained by MS^4^ of *m/z* 612 (data not shown). Thus, the MS^2^, MS^3^ and MS^4^ spectral features suggested that fraction N2-IX contained a branched dodecasaccharide with HexNAc-(Fuc-)Hex-HexNAc-(HexNAc-(Fuc-)Hex-HexNAc-)Hex-HexNAc-Hex-Hex sequence, and type 2 core on at least one of the HexNAc-(Fuc-)Hex-HexNAc branches. However, the binding of *H. pylori* strain J99 with generalist BabA to the slow-migrating dodecaosylceramide in fraction N-IX (Fig. 7c) indicated the presence of a type 1 core chain, since this variant of *H. pylori* BabA adhesin does not bind to blood group A or B determinants on type 2 core [12, 16]. Thus, fraction N-IX either contained two dodecaosylceramides with blood group A determinants on type 1 and type 2 core branches, or one dodecaosylceramide with blood group A determinants on one type 1 and one type 2 core branch.

In summary, four glycosphingolipids with terminal blood group A determinants (A tetraosylceramide, A type 1 hexaosylceramide, A type 1 octaosylceramide, and A dodecaosylceramide) were tentatively identified by chromatogram binding assays and mass spectrometry.

#### Characterization of the acid glycosphingolipids of moose I small intestine

##### Chromatogram binding assays

**Fig. 8:**
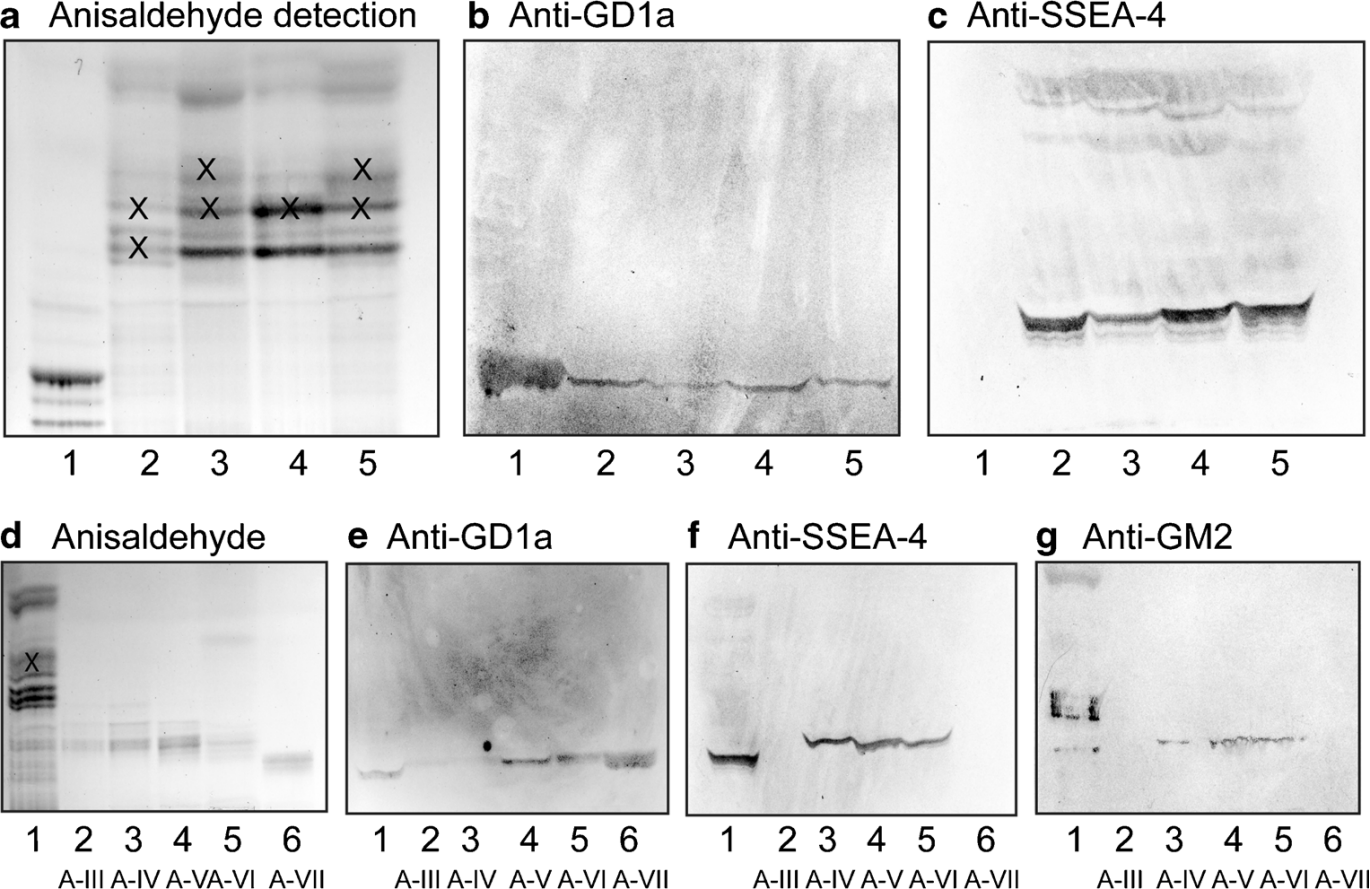
Binding of monoclonal antibodies to the acid glycosphingolipids from moose small and large intestine. Thin-layer chromatogram after detection with anisaldehyde (**a** and **d**), and autoradiograms obtained by binding of antibodies directed against the GD1a ganglioside (**b** and **e**), the sialyl-globopenta/SSEA-4 determinant (**c** and **f**) and the GM2 ganglioside (**g**). The glycosphingolipids were separated on aluminum-backed silica gel plates, using chloroform/methanol/water 60:35:8 (by volume) as solvent system, and the binding assays were performed as described under “Materials and methods”. Autoradiography was for 12 h. The lanes on **a**–**c** were: Lane 1, calf brain gangliosides, 40 μg; Lane 2, total acid glycosphingolipids of moose I small intestine, 40 μg; Lane 3, total acid glycosphingolipids of moose II small intestine, 40 μg; Lane 4, total acid glycosphingolipids of moose III small intestine, 40 μg; Lane 5, total acid glycosphingolipids of moose I large intestine, 40 μg. The lanes on **d**–**g** were: Lane 1, total acid glycosphingolipids of moose I small intestine, 40 μg; Lanes 2–6, fractions A-III – A-VII, 4 μg/lane. The bands marked with X on (**a**) and (**d**) are non-glycosphingolipid contaminants

Probing of the total acid glycosphingolipid fractions from the moose intestines with monoclonal anti-GD1a antibodies gave binding to all fractions (Fig. 8b). Also the monoclonal antibody directed against the SSEA-4 epitope gave a distinct binding to all fractions (Fig. 8c).

Binding of anti-SSEA-4 and anti-GD1a antibodies to the acid subfractions isolated from the small intestine of moose I demonstrated that the main anti-GD1a binding activity was to fractions A-V – A-VII (Fig. 8e), while the anti-SSEA-4 antibodies mainly bound to fractions A-IV – A-VI (Fig. 8f).

##### Mass spectrometry of the acid glycosphingolipids from moose I small intestine

The base peak chromatogram obtained by LC-ESI/MS of the total acid glycosphingolipid fraction from moose I small intestine (Supplementary Figure 2A) was dominated by molecular ions at *m/z* 794 and *m/z* 924, indicating sulfatide with d18:1-h16:0 and t18:0-h24:0 ceramide, respectively. MS^2^ of these molecular ions gave a B_1_ ion at *m/z* 241 and a C_1_ ion at *m/z* 259, confirming a SO_3_-Hex terminal (exemplified in Supplementary Figure 2B). Both MS^2^ spectra also had ions at *m/z* 540 and *m/z* 522, which are due to loss of the fatty acyl from the molecular ion [17]. The base peak chromatogram also had a minor molecular ion at *m/z* 1151. MS^2^ of this ion gave a series of Y ions (Y_0_ at *m/z* 536, Y_1_ at *m/z* 698, and Y_2_ at *m/z* 860), demonstrating a glycosphingolipid with NeuAc-Hex-Hex carbohydrate sequence and d18:1–16:0 ceramide, as the NeuAc-GM3 ganglioside (Supplementary Figure 2C). However, no other gangliosides could be identified by analysis of the total acid glycosphingolipid fraction.

Thus, in order to obtain sequence information of the slow-migrating gangliosides of moose I small intestine, the total acid glycosphingolipid fraction was separated into seven subfractions. LC-ESI/MS and MS^2^ of fraction A-I identified sulfatide with d18:1-h16:0 and t18:0-h24:0, as above, while fraction A-II had two predominant molecular ions at *m/z* 1286 and *m/z* 1314. NeuGc-GM3 with t18:0-h22:0 and t18:0-h24:0 was identified by MS^2^ of the latter two ions (data not shown).

##### Fractions A-III and A-VII

The base peak chromatograms from LC-ESI/MS of fractions A-III and A-VII (Supplementary Figure 3A and B) both had a doubly charged molecular ion at *m/z* 917, eluting at 26.7–32.3 min and 27.9–31.9 min, respectively. This indicated gangliosides with two NeuAc, one HexNAc, three Hex, and d18:1–18:0 ceramide, as the GD1a or GD1b gangliosides. The MS^2^ spectra obtained were in both cases relatively weak. However, MS^2^ of the ion at *m/z* 917 of fraction A-III (Supplementary Figure 3C) gave a B_2_ ion at *m/z* 581, demonstrating a NeuAc-NeuAc sequence, and a series of Y ions (Y_1_ at *m/z* 726, Y_2_ at *m/z* 888, Y_3α_ at *m/z* 1091, and Y_3β_ at *m/z* 1254). Taken together this demonstrated a ganglioside with Hex-HexNAc-(NeuAc-NeuAc-)Hex-Hex sequence, as GD1b [18]. This conclusion was supported by the absence of binding of the anti-GD1a antibodies to fraction A-III (Fig. 8e, lane 2).

In contrast, a terminal NeuAc-Hex-HexNAc sequence was demonstrated by the C_2α_ ion at *m/z* 470 and the B_3α_ ion at *m/z* 655 in the MS^2^ spectrum obtained of the ion at *m/z* 917 of fraction A-VII (Supplementary Figure 3D). Taken together with the binding of the anti-GD1a antibodies to fraction A-VII (Fig. 8e, lane 6), this gave a tentative identification of a ganglioside with NeuAc-Hex-HexNAc(NeuAc)Hex-Hex sequence and d18:1–18:0 ceramide, *i.e.,* the GD1a ganglioside [18].

##### Fractions A-IV - A-VI

**Fig. 9:**
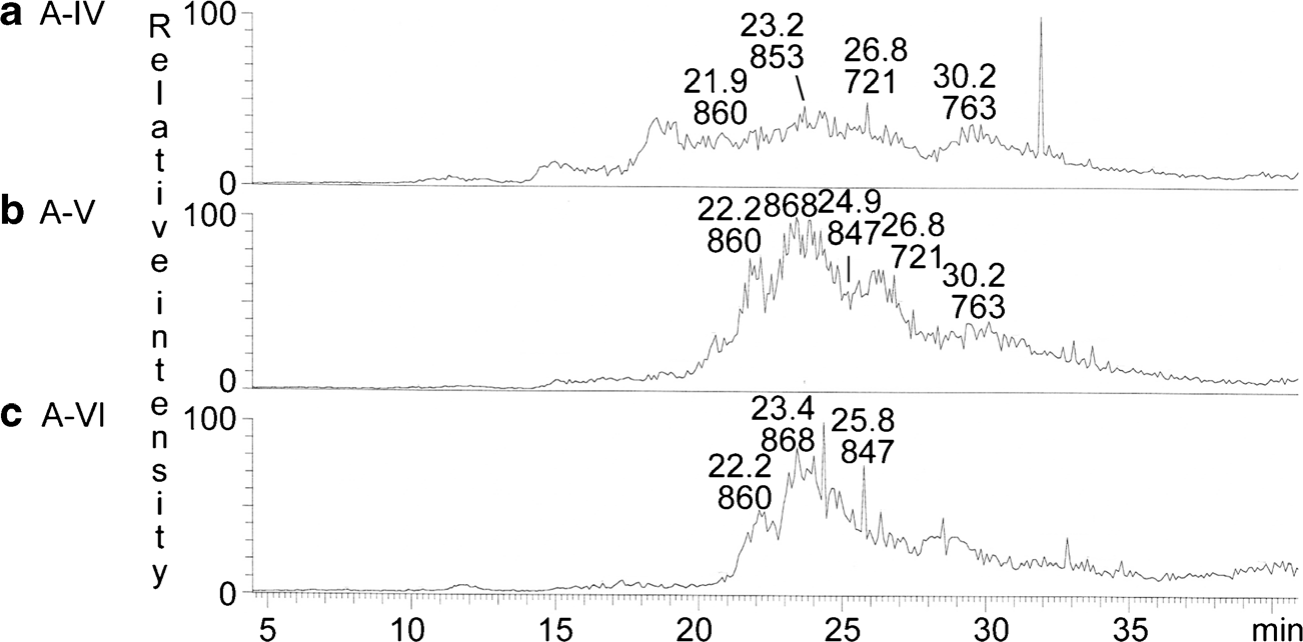
LC-ESI/MS of the acid glycosphingolipids from moose I small intestine. **a** Base peak chromatogram from LC-ESI/MS of fraction A-IV from moose I small intestine. **b** Base peak chromatogram from LC-ESI/MS of fraction A-V from moose I small intestine. **c** Base peak chromatogram from LC-ESI/MS of fraction A-VI from moose I small intestine

The base peak chromatograms of fractions A-IV, A-V and A-VI showed that these three fractions all contained several compounds (Fig. 9). Both fractions A-IV and A-V had doubly charged molecular ions at *m/z* 721 and *m/z* 763, both corresponding to a ganglioside with two NeuAc and two Hex, having d18:1–16:0 and d18:1–22:0 ceramide, respectively. MS^2^ of the molecular ion at *m/z* 721 (data not shown) gave two B ions (B_1_ at *m/z* 290 and B_2_ at *m/z* 581) demonstrating a terminal NeuAc-NeuAc sequence. In addition, there was a series of Y ions (Y_0_ at *m/z* 536, Y_2_ at *m/z* 860, and Y_3_ at *m/z* 1151). Thus, a ganglioside with NeuAc-NeuAc-Hex-Hex sequence and d18:1–16:0 ceramide, as the NeuAc-GD3 ganglioside, was indicated. MS^2^ of the doubly charged molecular ion at *m/z* 763 also identified a NeuAc-GD3 ganglioside, in this case with d18:1–22:0 ceramide (data not shown).

**Fig. 10:**
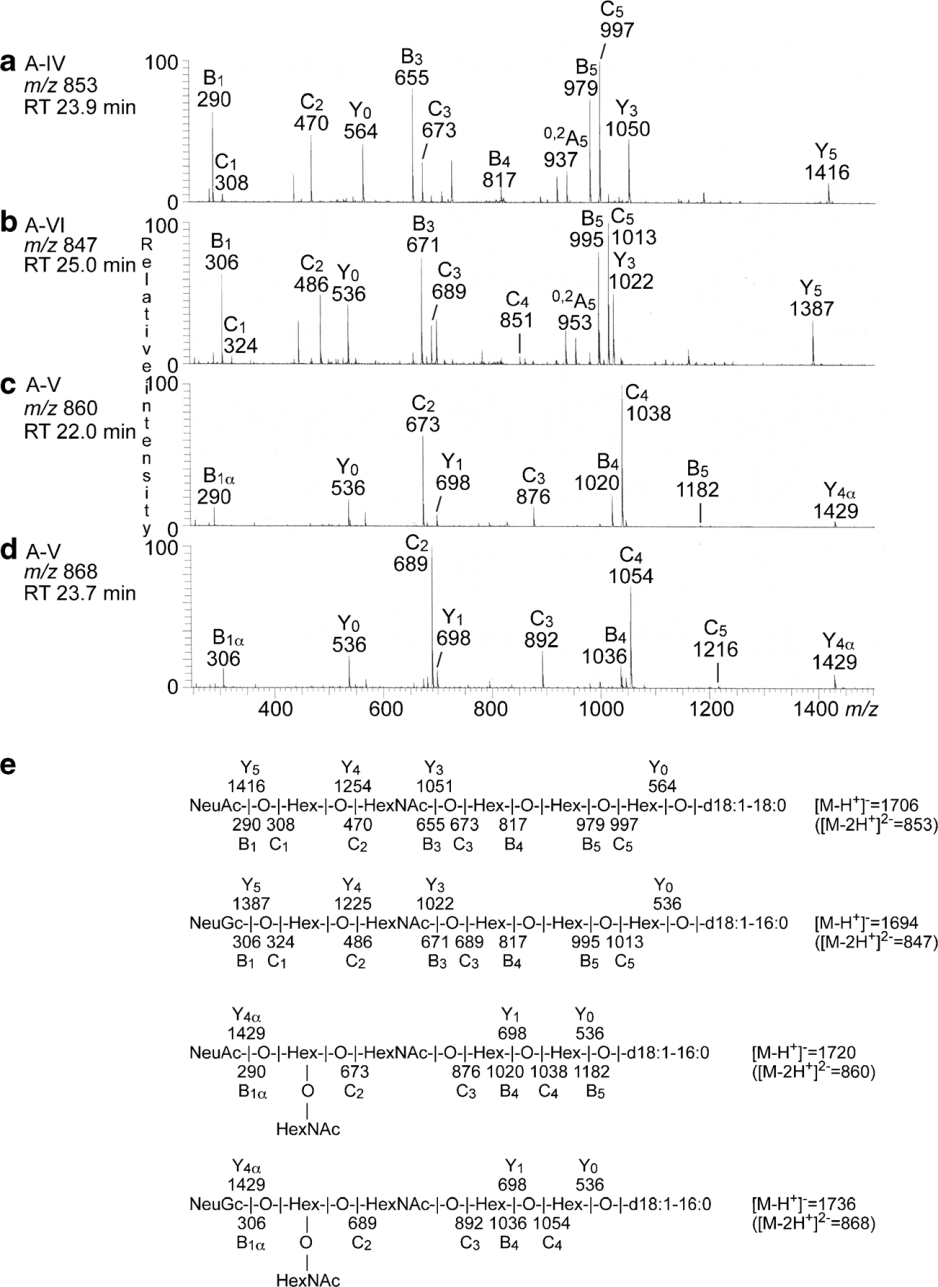
LC-ESI/MS of the acid glycosphingolipid fractions from moose I small intestine. **a** MS^2^ of the ion at *m/z* 853 (retention time 23.9 min) from LC-ESI/MS of fraction A-IV. **b** MS^2^ of the ion at *m/z* 847 (retention time 25.0 min) from LC-ESI/MS of fraction A-VI. **c** MS^2^ of the ion at *m/z* 860 (retention time 22.0 min) from LC-ESI/MS of fraction A-V. **d** MS^2^ of the ion at *m/z* 868 (retention time 23.7 min) from LC-ESI/MS of fraction A-V. **e** Interpretation formulas showing the deduced carbohydrate sequences and ceramide composition

The doubly charged molecular ion at *m/z* 853 in fraction A-IV indicated a ganglioside with one NeuAc, one HexNAc, four Hex, and d18:1–18:0 ceramide, while the doubly charged molecular ion at *m/z* 847 in fractions A-V and A-VI indicated a ganglioside with one NeuGc, one HexNAc, four Hex, and d18:1–16:0 ceramide. MS^2^ of the ion at *m/z* 853 (Fig. 10a) gave B and C type fragment ions (B_1_ at *m/z* 290, C_1_ at *m/z* 308, C_2_ at *m/z* 470, B_3_ at *m/z* 655, C_3_ at *m/z* 673, B_4_ at *m/z* 817, B_5_ at *m/z* 979, and C_5_ at *m/z* 997) demonstrating a NeuAc-Hex-HexNAc-Hex-Hex-Hex carbohydrate sequence. The Y_3_ ion at *m/z* 1050 was further evidence for a Hex-Hex-Hex sequence at the reducing end, and the ^0,2^A_5_ ion at *m/z* 937 indicated a 4-substitution of the second Hex from the reducing end.

The same carbohydrate sequence but with NeuGc instead of NeuAc, *i.e.,* a NeuGc-Hex-HexNAc-Hex-Hex-Hex sequence, was indicated by MS^2^ of the molecular ion at *m/z* 847 in fraction A-V and A-VI (Fig. 10b). This was concluded from the B and C type fragment ions series (B_1_ at *m/z* 306, C_1_ at *m/z* 324, C_2_ at *m/z* 486, B_3_ at *m/z* 671, C_3_ at *m/z* 689, C_4_ at *m/z* 851, B_5_ at *m/z* 995, and C_5_ at *m/z* 1013). Here, the Y_3_ ion at *m/z* 1022 was evidence for a Hex-Hex-Hex sequence at the reducing end, and the ^0,2^A_5_ ion at *m/z* 953 indicated that the second Hex from the reducing end was substituted at C-4.

Thus, MS^2^ indicated NeuAc and NeuGc variants of a ganglioside with NeuAc/NeuGc-Hex-HexNAc-Hex-Hex-Hex sequence, most likely sialyl-globopentaosylceramide (SSEA-4 ganglioside). This suggestion was corroborated by the binding of anti-sialyl-globopenta/SSEA-4 antibodies to fractions A-IV, A-V and A-VI (Fig. 8f).

The doubly charged molecular ions at *m/z* 860 in fractions A-IV – A-VI indicated a ganglioside with one NeuAc, two HexNAc, and three Hex, and d18:1–16:0, while the molecular ion at *m/z* 868 fractions A-V and A-IV suggested a ganglioside with one NeuGc, two HexNAc, and three Hex, and d18:1–16:0.

The MS^2^ spectrum of the ion at *m/z* 860 (Fig. 10c) had a number of B- and C-type fragment ions (B_1_ at *m/z* 290, C_2_ at *m/z* 673, C_3_ at *m/z* 876, B_5_ at *m/z* 1020, C_4_ at *m/z* 1038, and B_5_ at *m/z* 1182) demonstrating a NeuAc-(HexNAc + Hex)-HexNAc-Hex-Hex sequence, and the MS^2^ spectrum of the ion at *m/z* 868 (Fig. 10d) had the corresponding B- and C-type fragment ions (B_1_ at *m/z* 306, C_2_ at *m/z* 689, C_3_ at *m/z* 892, C_4_ at *m/z* 1054, and C_5_ at *m/z* 1216) demonstrating a NeuGc-(HexNAc + Hex)-HexNAc-Hex-Hex sequence. No B_2_ or C_2_ ions were obtained by MS^2^ or MS^3^, so the order of the penultimate HexNAc and Hex could not be determined.

Thus, MS^2^ of the molecular ions at *m/z* 860 and *m/z* 868 demonstrated NeuAc and NeuGc variants of a ganglioside with one NeuAc or NeuGc, two HexNAc and three Hex. Of previously identified gangliosides there are three alternatives. Firstly, the GalNAc-GM1 ganglioside (GalNAcβ3Galβ3GalNAcβ4(NeuAc/NeuGcα3)Galβ4Glcβ1Cer). However, this alternative is not likely since the C_3_ ions at *m/z* 673 and *m/z* 689 demonstrated a terminal NeuAc-Hex-HexNAc sequence and a terminal NeuGc-Hex-HexNAc sequence, respectively. The second alternative is the NeuAc-*x*_2_ ganglioside (NeuAcα3GalNAcβ3Galβ4GlcNAcβ3Galβ4Glcβ1Cer) and the third is the Sd^a^ ganglioside (NeuAcα3(GalNAcβ4)Galβ4GlcNAcβ3Galβ4Glcβ1Cer). MS^3^ gave no B_2_ or C_2_ ions that allowed differention between these two alternatives. However, the binding of anti-GM2 antibodies in the slow-migrating region of fractions A-IV – A-VI (Fig. 8g) supported a Sd^a^ ganglioside, since these antibodies recognize a terminal NeuAcα3(GalNAcβ4)Gal sequence.

##### Comparison with fallow deer intestine

Acid and non-acid glycosphingolipids were also isolated from mucosal scrapings of fallow deer small intestine, with the intention of comparing with the intestinal glycosphingolipid profile of a species related to the moose. The major compound of the non-acid fraction of fallow deer intestine was monoglycosylceramide. By separation into subfractions, and characterization of these with chromatogram binding assays and mass spectrometry as above, the two major complex non-acid glycosphingolipids were characterized as Galili penta- and heptaosylceramide (data not shown). However, no glycosphingolipids with terminal blood group A/B/H determinants were found.

The acid glycosphingolipid fractions of moose small intestine and fallow deer small intestine were very similar. Thus, sulfatide, and the gangliosides GM3, GD3, GD1a and GD1b, were also characterized in the case of fallow deer intestine, using the same strategies as for moose intestine (data not shown).

**Fig. 11:**
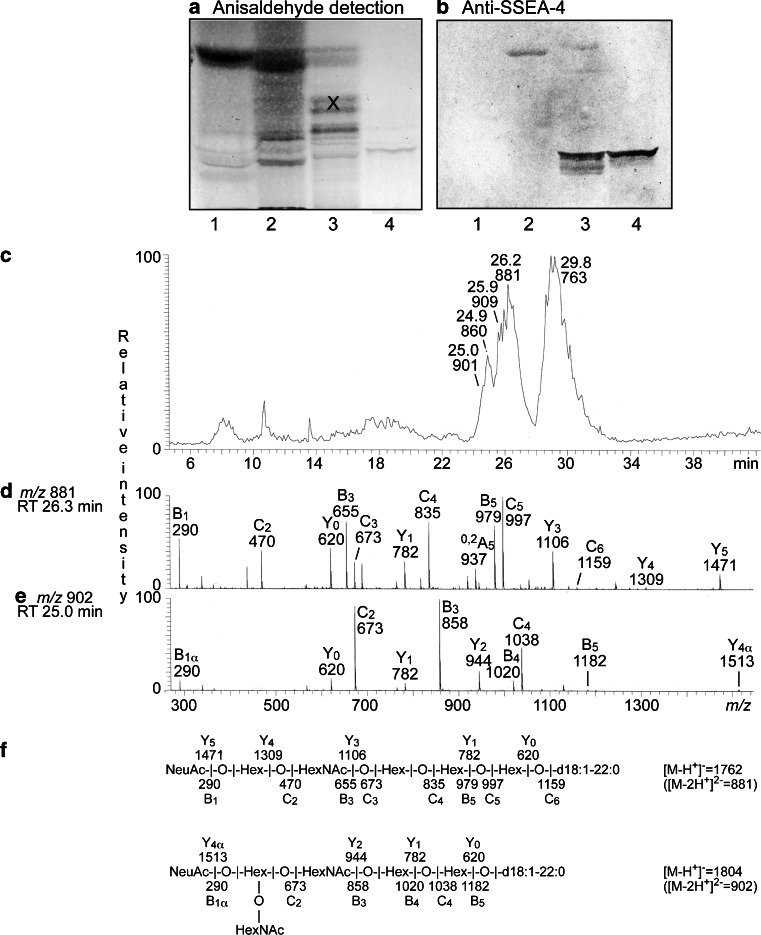
Comparison of acid glycosphingolipids from horse, bovine, and moose small intestine. Thin-layer chromatogram after detection with anisaldehyde (**a**), and autoradiogram obtained by binding antibodies directed against the sialyl-globopenta/SSEA-4 determinant (**b**). The glycosphingolipids were separated on aluminum-backed silica gel plates, using chloroform/methanol/water 60:35:8 (by volume) as solvent system, and the binding assays were performed as described under “Materials and methods”. Autoradiography was for 12 h. The lanes were: Lane 1, total acid glycosphingolipids of horse small intestine, 40 μg; Lane 2, total acid glycosphingolipids of bovine small intestine, 40 μg; Lane 3, total acid glycosphingolipids of moose small intestine, 40 μg; Lane 1, acid glycosphingolipid subfraction (fraction FD-VI) isolated from fallow deer small intestine, 4 μg. The bands marked with an X in lane 3 are non-glycosphingolipid contaminants. **c** Base peak chromatogram from LC-ESI/MS of fraction fraction FD-VI from fallow deer small intestine. **d** MS^2^ of the ion at *m/z* 881 (retention time 26.3 min) from LC-ESI/MS of fraction FD-VI. **e** MS^2^ of the ion at *m/z* 902 (retention time 25.0 min) from LC-ESI/MS of fraction FD-VI. **f** Interpretation formulas showing the deduced carbohydrate sequences and ceramide composition

Furthermore, a subfraction with distinct binding of anti-SSEA-4 antibodies was obtained upon separation of the acid fraction from fallow deer intestine (Fig. 11b, lane 4). LC-ESI/MS of this fraction gave a base peak chromatogram with a number of doubly charged molecular ions at *m/z* 763, *m/z* 860, *m/z* 881, *m/z* 901 and *m/z* 909 (Fig. 11c). The doubly charged molecular ion at *m/z* 881 indicated a ganglioside with one NeuAc, one HexNAc, four Hex, and d18:1–22:0 ceramide. A NeuAc-Hex-HexNAc-Hex-Hex-Hex carbohydrate sequence was demonstrated by the B and C type fragment ions (B_1_ at *m/z* 290, C_2_ at *m/z* 470, B_3_ at *m/z* 655, C_3_ at *m/z* 673, C_4_ at *m/z* 835, B_5_ at *m/z* 979, C_5_ at *m/z* 997, and C_6_ at *m/z* 1159) obtained by MS^2^ (Fig. 11d). The features of this MS^2^ spectrum were very similar to the MS^2^ spectrum of moose intestinal sialyl-globopentaosylceramide/SSEA-4 (Fig. 10a), allowing identification of this ganglioside also in fallow deer intestine.

In a similar manner, a NeuAc-Sd^a^ ganglioside with d18:1–22:0 ceramide was identified by MS^2^ of the ion at *m/z* 902 (Fig. 11e). This was concluded from the series of B and C type fragment ions obtained by MS^2^ (B_1_ at *m/z* 290, C_2_ at *m/z* 673, B_3_ at *m/z* 858, B_5_ at *m/z* 1020, C_4_ at *m/z* 1038, and B_5_ at *m/z* 1182) demonstrating a NeuAc-(HexNAc + Hex)-HexNAc-Hex-Hex sequence. Furthermore, a NeuAc-Sd^a^ ganglioside with d18:1–16:0 ceramide was found by MS^2^ of the ion at *m/z* 860, and a NeuGc-Sd^a^ ganglioside with d18:1–22:0 ceramide found by MS^2^ of the ion at *m/z* 909. Finally, MS^2^ of the ion at *m/z* 763 identified a NeuAc-GD3 ganglioside with d18:1–22:0 ceramide (data not shown).

## Discussion

Moose are the largest land mammals in the circumpolar boreal forests of Eurasia and Canada, and are an important game species. Their diet consists of browse, tree, and shrub leaves and stems, and like all ruminants, the moose gastrointestinal tract has a four chambered stomach, in addition to the small and large intestine. In this study the non-acid and acid glycosphingolipids of moose small and large intestine were characterized. The complex non-acid glycosphingolipid fractions of the three small intestines, and the one large intestine, all had Galili penta- and heptaglycosylceramides. In addition, two of the moose small intestines, and the one large intestine, had glycosphingolipids with terminal blood group O determinants (H triglycosylceramide, H type 2 pentaosylceramide, H type 1 penta- and heptaosylceramide), while glycosphingolipids with terminal blood group A determinants (A tetraosylceramide, A type 1 hexa- and octaosylceramide, A dodecaosylceramide), were found in the third moose small intestine.

Glycosphingolipids with terminal histo-blood group A, B or O determinants are present in the gastrointestinal tract of many different species [2]. The types of ABO determinants varies depending on species, and also between individuals of the same species. Thus, in the human small intestinal epithelium the expression of glycosphingolipids with terminal blood group A, B or O determinants varies depending on the ABO blood group of the individual donors [19]. However, in pigs there are only blood group A and O determinants [20]. Here, the H type 1 pentaglycosylceramide is present in the small intestinal epithelium of blood group O pigs, while blood group A pigs have the A type 1 hexaglycosylceramide, the A type 4 heptaglycosylceramide, the A type 1 octaglycosylceramide, and a repetitive A type 1 nonaglycosylceramide [21].

In this study we demonstrate that glycosphingolipids with terminal blood group H determinants are present in the small intestine of certain moose individuals, whereas other moose individuals have glycosphingolipids with terminal blood group A determinants. However, due to the small number of moose intestines characterized, it can not be excluded that there are also moose with intestinal glycosphingolipids with blood group B determinants.

In addition to the glycosphingolipids with blood group determinant, all the intestinal samples had glycosphingolipids belonging to the lacto- and neolactoseries (lactotriaosylceramide, lactotetraosylceramide, neolactotetraosylceramide, Galα3-Le^x^ hexaosylceramide, and lacto-neolactohexaosylceramide), globo-series (globotriaosylceramide and globotetraosylceramide), and isogloboseries (isoglobotriaosylceramide). Thus, moose intestine is a good source for isolation of variant glycosphingolipids.

The chemical staining of the total non-acid fractions separated on thin-layer chromatograms shows that the tri- and tetraglycosylceramides are major compounds. Yet, in the base peak chromatograms from LC/ESI-MS of the oligosaccharides released from the glycosphingolipids, the molecular ions corresponding to tri- and tetrasaccharides (at *m/z* 503, 544 and 706) are relatively minor. This discrepancy is due to the low capacity of the *Rhodococcus* endoglycoceramidase for hydrolysis of globo-series glycosphingolipids [22, 23]. The ideal enzyme would have been the ceramide glycanase from *Macrobdella decora*, which has a more universal hydrolytic activity towards glycosphingolipids [24]. However, this enzyme is no longer available on the market. In fact, in certain cases, as when analyzing total non-acid glycosphingolipid mixtures with high amounts of globotri- and globotetraosylceramide, this relative resistance *Rhodococcus* endoglycoceramidase might be an advantage allowing detection of minor complex glycosphingolipids.

Acid fractions of moose small and large intestine were very similar, *i.e.,* all contained sulfatide, and the gangliosides GM3, GD3, GD1a, GD1b, and also the Sd^a^ ganglioside and sialyl-globopentaosylceramide/SSEA-4. Interestingly, the same acid glycosphingolipids were present in fallow deer small intestine. However, no binding of the anti-SSEA-4 antibodies to the acid glycosphingolipids of horse and bovine small intestine occurred, indicating that sialyl-globopentaosylceramide was not present in these species. This is consistent with previous characterization of the acid glycosphingolipids of adult bovine small intestinal epithelium were sulfatide, NeuAc-/NeuGc-GM3, NeuAc-/NeuGc-GM2, NeuAc-/NeuGc-GM1, and Gc-GD2 were found [25].

The Sd^a^ determinant is expressed on epithelial glycosphingolipids and glycoproteins, and mucins in the normal gastrointestinal tract in the majority of humans, and in several other species [26]. The structure of the Sd^a^ determinant was first determined as the GalNAcβ4(NeuAcα3)Galβ4GlcNAcβ3Gal pentasaccharide carried by N-glycans of the Tamm-Horsfall glycoprotein [27]. The Sd^a^ antigen is related to, but not entirely identical with, the Cad antigen. The Cad antigen was first characterized as the O-linked pentasaccharide GalNAcβ4(NeuAcα3)Galβ3(NeuAcα6)GalNAc of glycophorin A [28]. Thus, the two antigens have the same terminal trisaccharide, GalNAcβ4(NeuAcα3)Galβ. Interestingly, a number of different physiological and pathological roles of the Sd^a^ determinant have been described (reviewed in [29]). Thus, in the murine system the Sd^a^ antigen plays a role in the lytic function of cytotoxic T lymfocytes, is involved in the regulation of the circulating half-life of the von Willebrand factor and also in implantation of embryos to the endometrium. Furthermore, overexpression of the Sd^a^ determinant in skeletal muscle fibers gives a reversion of dystrophy in murine models of muscular dystrophy. Finally, in humans the Sd^a^ antigen is down-regulated in gastric and colon cancer.

The finding of sialyl-globopentaosylceramide/SSEA-4, with both *N*-acetyl and *N*-glycolyl neuraminic acid, in moose intestine is to our knowledge the first time sialyl-globopentaosylceramide/SSEA-4 has been characterized in a normal fully differentiated tissue, and also the first time NeuGc-globopentaosylceramide has been characterized. NeuAc-globopentaosylceramide carries the determinant of the stage specific embryonic antigen 4 (SSEA-4). The stage specific embryonic antigens were originally identified by three monoclonal antibodies (SSEA-1, SSEA-3, and SSEA-4) prepared against murine embryos [30], and recognize defined carbohydrate epitopes associated with the lacto- and globo-series glycosphingolipids [31–33]. In humans SSEA-4 is a marker for embryonic and adult stem cells [34–36]. Upon differentiation of human embryonic stem cells, the expression of SSEA-4 is rapidly diminished, and has almost completely disappeared after 14 days [37, 38], *i.e.,* SSEA-4 downregulation accompanies the loss of pluripotency.

According to the cancer stem cells theory [39], cancers are maintained by subpopulations of tumor cells with stem or progenitor cell characteristics expressing stem cell associated markers, and it are these cells that initiate tumor formation and differentiate along multi-potent pathways. In agreement with this theory, SSEA-4 is also expressed in several human cancers, as *e.g.,* breast cancer [40], ovarian carcinoma [41], renal cell carcinoma [42], basaloid lung cancer [43], and glioblastoma [44].

However, in the present study the sialyl-globopenta/SSEA-4 ganglioside was characterized in moose small and large intestine, and also in fallow deer small intestine, *i.e.,* in normal tissues of adult individuals. This once again demonstrates that the one carbohydrate structure may have different biological roles in different species, at different times in development and in different tissues.

## Electronic supplementary material

Supplementary Fig. 1 LC-ESI/MS of oligosaccharides obtained by digestion of the non-acid glycosphingolipid fractions N2-IV and N2-VII from moose II intestine with *Rhodococcus* endoglycoceramidase II. (A) Base peak chromatogram from LC-ESI/MS of the oligosaccharides derived from fraction N2-IV from moose II small intestine. (B) Base peak chromatogram from LC-ESI/MS of the oligosaccharides derived from fraction N2-VII from moose II small intestine. The identification of individual glycosphingolipid-derived oligosaccharides given in chart A and B was based on their determined molecular masses and subsequent MS^2^ sequencing. A4, GalNAcα3(Fucα2)Galβ4Glc; Gb3, Galα4Galβ4Glc; Gb4, GalNAcβ3Galα4Galβ4Glc; H3, Fucα2Galβ4Glc; nLc4, Galβ4GlcNAcβ3Galβ4Glc; A6-1, GalNAcα3(Fucα2)Galβ3GlcNAcβ3Galβ4Glc; A8-1, GalNAcα3(Fucα2)Galβ3GlcNAcβ3Galβ3GlcNAcβ3Galβ4Glc; B5, Galα3Galβ4GlcNAcβ3Galβ4Glc; B7, Galα3Galβ4GlcNAcβ3Galβ4GlcNAcβ3Galβ4Glc. (C) MS^2^ of the ion at *m/z* 690 (retention time 19.6 min) from LC-ESI/MS of fraction N2-IV. (D) MS^2^ of the ion at *m/z* 1055 (retention time 24.9 min) from LC-ESI/MS of fraction N2-VII. (E) MS^2^ of the ion at *m/z* 1420 (retention time 29.9 min) from LC-ESI/MS of fraction N2-VII. (F) Interpretation formulas showing the deduced carbohydrate sequences. (PDF 772 kb)

Supplementary Fig. 2 LC-ESI/MS of the total acid glycosphingolipid fraction from moose I small intestine. (A) Base peak chromatogram from LC-ESI/MS of the total acid glycosphingolipid fraction from moose I small intestine. (B) MS^2^ of the ion at *m/z* 794 (retention time 3.4 min). (C) MS^2^ of the ion at *m/z* 1151 (retention time 12.9 min). (D) Interpretation formulas showing the deduced glycosphingolipid structures. (PDF 452 kb)

Supplementary Fig. 3 LC-ESI/MS of the acid glycosphingolipid fractions A-III and A-VII from moose I small intestine. (A) Base peak chromatogram from LC-ESI/MS of fraction A-III from moose I intestine. (B) Base peak chromatogram from LC-ESI/MS of fraction A-VII from moose I intestine. (C) MS^2^ of the ion at *m/z* 917 (retention time 28.0 min) from ESI/MS of fraction A-III. (D) MS^2^ of the ion at *m/z* 917 (retention time 29.2 min) from ESI/MS of fraction A-VII. (E) Interpretation formulas showing the deduced glycosphingolipid structures. (PDF 626 kb)

## Abbreviations

LC-ESI/MS: Liquid chromatography/electrospray ionization mass spectrometry
SSEA: Stage specific embryonic antigen
