# Supplementary material for: Characterization of moose intestinal glycosphingolipids
Source: Glycoconj J. 2015 Jun 24;32(6):393–412. doi: 10.1007/s10719-015-9604-8 (PMC4515253; doi:10.1007/s10719-015-9604-8)
Supplement: Supplementary file 1 — LC-ESI/MS of oligosaccharides obtained by digestion of the non-acid glycosphingolipid fractions N2-IV and N2-VII from moose II intestine with Rhodococcus endoglycoceramidase II. (A) Base peak chromatogram from LC-ESI/MS of the oligosaccharides derived from fraction N2-IV from moose II small intestine. (B) Base peak chromatogram from LC-ESI/MS of the oligosaccharides derived from fraction N2-VII from moose II small intestine. The identification of individual glycosphingolipid-derived oligosaccharides given in chart A and B was based on their determined molecular masses and subsequent MS2 sequencing. A4, GalNAcα3(Fucα2)Galβ4Glc; Gb3, Galα4Galβ4Glc; Gb4, GalNAcβ3Galα4Galβ4Glc; H3, Fucα2Galβ4Glc; nLc4, Galβ4GlcNAcβ3Galβ4Glc; A6-1, GalNAcα3(Fucα2)Galβ3GlcNAcβ3Galβ4Glc; A8-1, GalNAcα3(Fucα2)Galβ3GlcNAcβ3Galβ3GlcNAcβ3Galβ4Glc; B5, Galα3Galβ4GlcNAcβ3Galβ4Glc; B7, Galα3Galβ4GlcNAcβ3Galβ4GlcNAcβ3Galβ4Glc. (C) MS2 of the ion at m/z 690 (retention time 19.6 min) from LC-ESI/MS of fraction N2-IV. (D) MS2 of the ion at m/z 1055 (retention time 24.9 min) from LC-ESI/MS of fraction N2-VII. (E) MS2 of the ion at m/z 1420 (retention time 29.9 min) from LC-ESI/MS of fraction N2-VII. (F) Interpretation formulas showing the deduced carbohydrate sequences. (PDF 772 kb) [file 10719_2015_9604_MOESM1_ESM.pdf]

Fig. S1

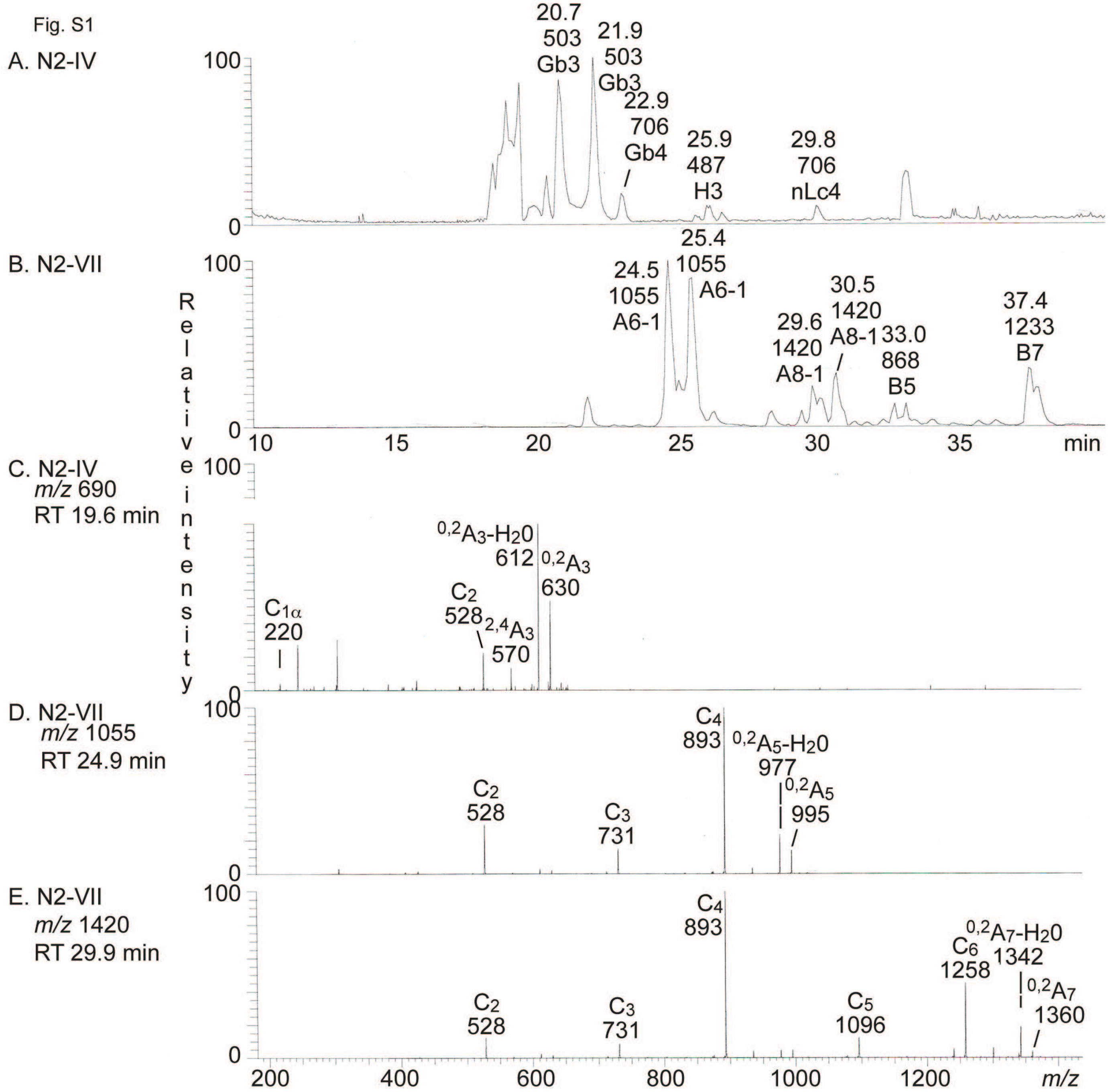

F.

```
HexNAc-|-O-|-Hex-|-O-|-Hex  [M-H+]-=690
     220 |     528
     C1α O     C2
         |
        Fuc

HexNAc-|-O-|-Hex-|-O-|-HexNAc-|-O-|-Hex-|-O-|-Hex  [M-H+]-=1055
         |      528          731        893
         O      C2           C3         C4
         |
        Fuc

HexNAc-|-O-|-Hex-|-O-|-HexNAc-|-O-|-Hex-|-O-|-HexNAc-|-O-|-Hex-|-O-|-Hex  [M-H+]-=1420
         |      528          731        893          1096       1258
         O      C2           C3         C4           C5         C6
         |
        Fuc
```
