# Supplementary figures and images for: Characterization of moose intestinal glycosphingolipids
Source: Glycoconj J. 2015 Jun 24;32(6):393–412. doi: 10.1007/s10719-015-9604-8 (PMC4515253; doi:10.1007/s10719-015-9604-8)

Fig. S2

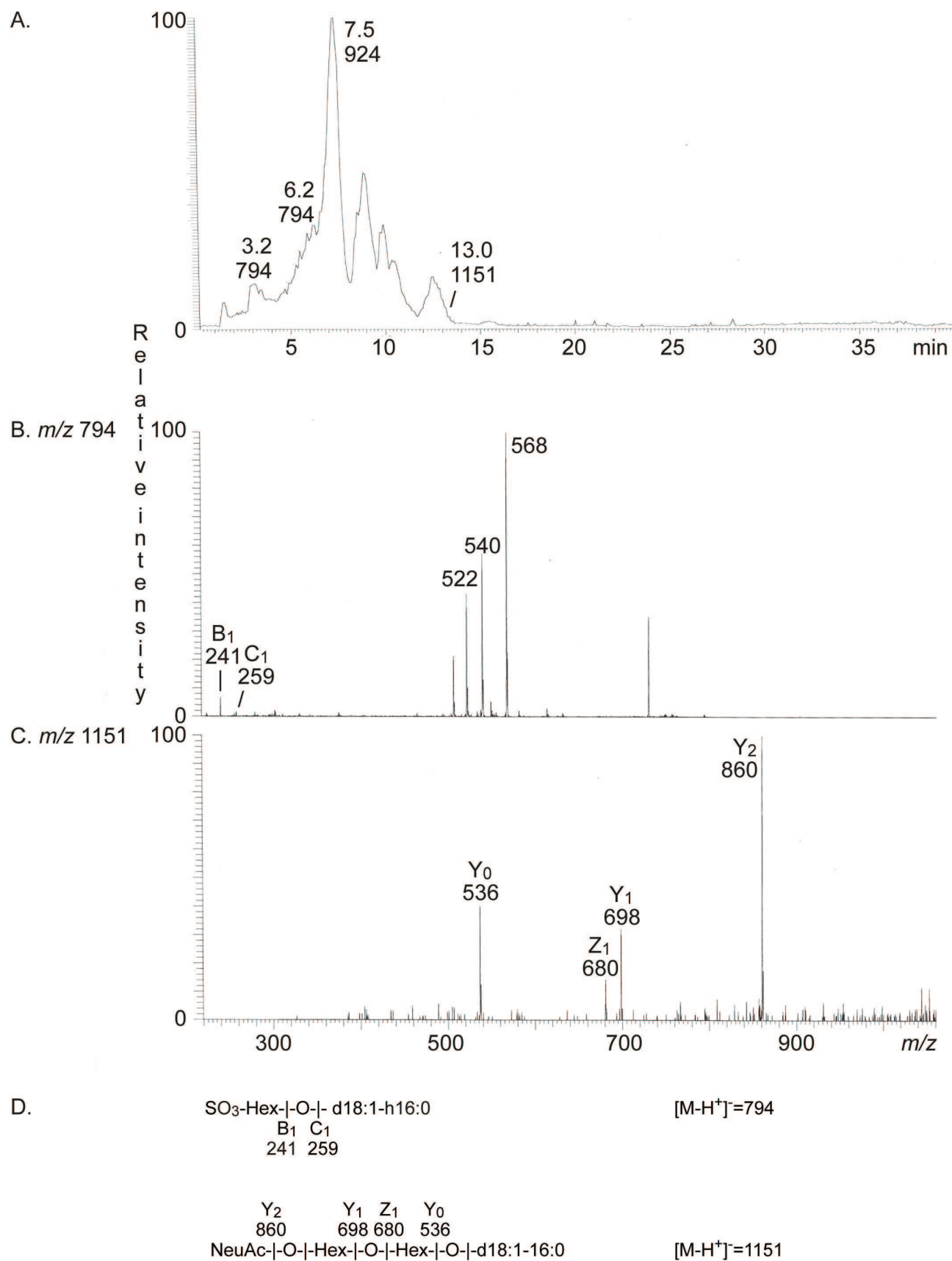

Supplement: Supplementary file 2 — LC-ESI/MS of the total acid glycosphingolipid fraction from moose I small intestine. (A) Base peak chromatogram from LC-ESI/MS of the total acid glycosphingolipid fraction from moose I small intestine. (B) MS2 of the ion at m/z 794 (retention time 3.4 min). (C) MS2 of the ion at m/z 1151 (retention time 12.9 min). (D) Interpretation formulas showing the deduced glycosphingolipid structures. (PDF 452 kb) [file 10719_2015_9604_MOESM2_ESM.pdf]

Fig. S3

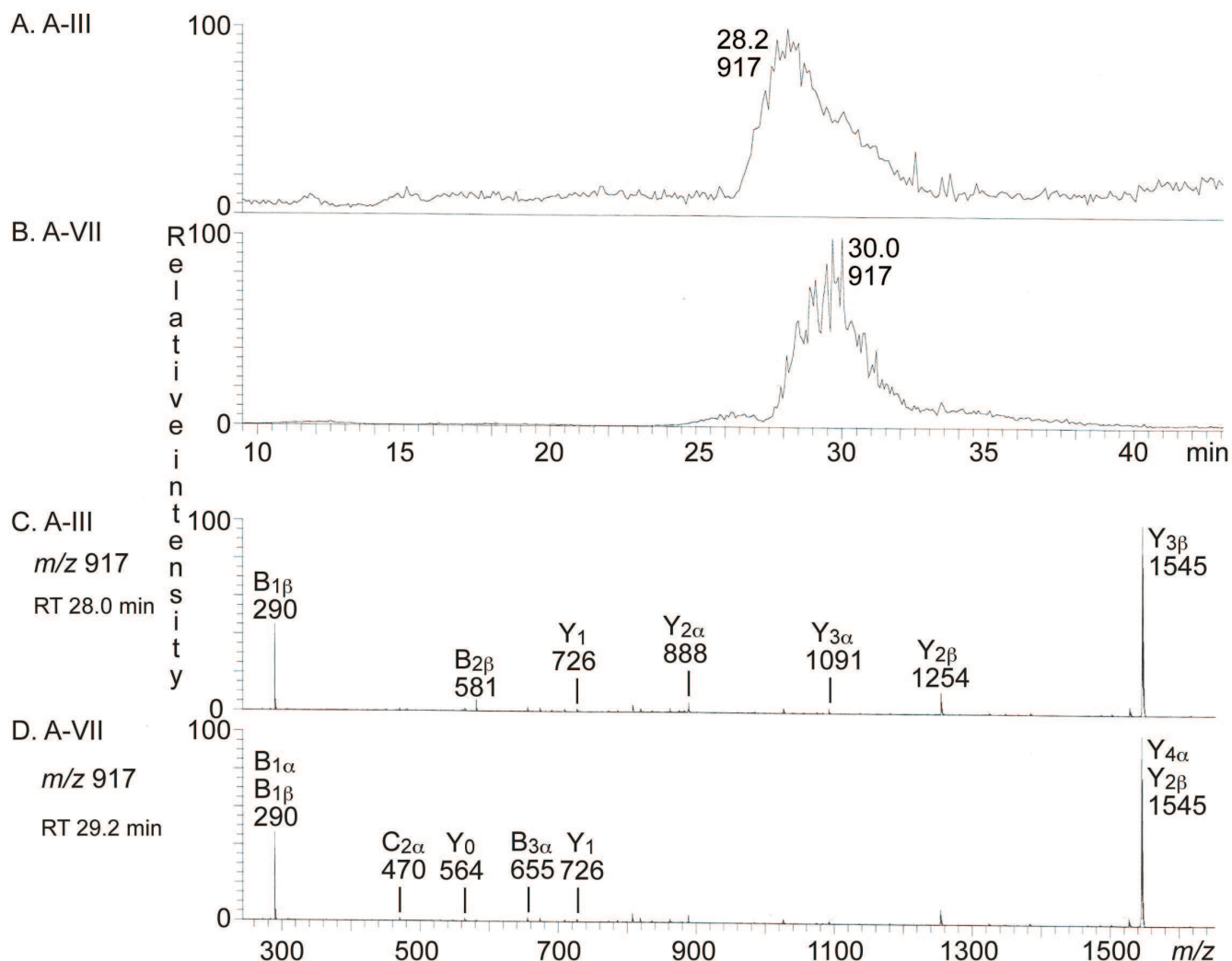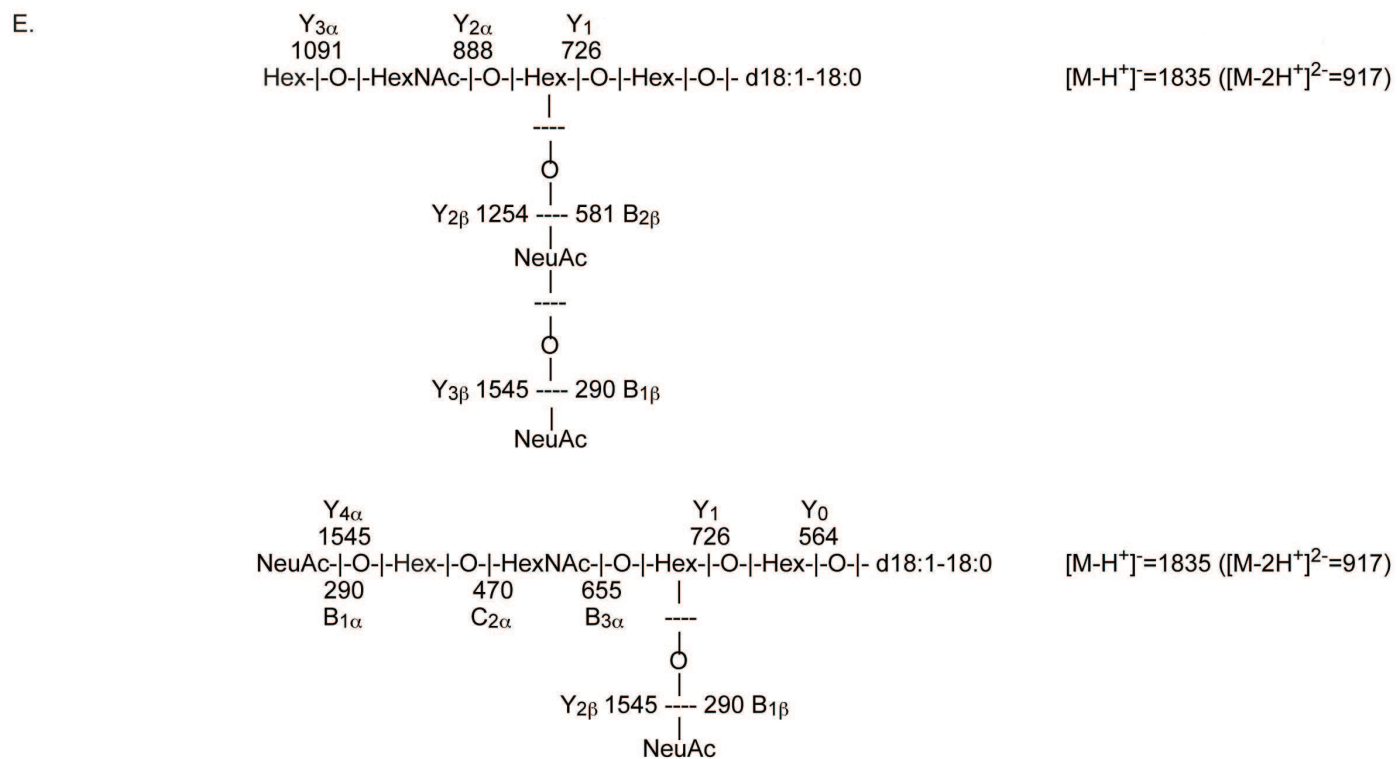

Supplement: Supplementary file 3 — LC-ESI/MS of the acid glycosphingolipid fractions A-III and A-VII from moose I small intestine. (A) Base peak chromatogram from LC-ESI/MS of fraction A-III from moose I intestine. (B) Base peak chromatogram from LC-ESI/MS of fraction A-VII from moose I intestine. (C) MS2 of the ion at m/z 917 (retention time 28.0 min) from ESI/MS of fraction A-III. (D) MS2 of the ion at m/z 917 (retention time 29.2 min) from ESI/MS of fraction A-VII. (E) Interpretation formulas showing the deduced glycosphingolipid structures. (PDF 626 kb) [file 10719_2015_9604_MOESM3_ESM.pdf]
